# Identification of Phenotypic Lipidomic Signatures in Response to Long Chain n‐3 Polyunsaturated Fatty Acid Supplementation in Humans

**DOI:** 10.1161/JAHA.120.018126

**Published:** 2021-01-19

**Authors:** Matthew Picklo, Bastien Vallée Marcotte, Michael Bukowski, Juan de Toro‐Martín, Bret M. Rust, Frédéric Guénard, Marie‐Claude Vohl

**Affiliations:** ^1^ USDA‐ARS Grand Forks Human Nutrition Research Center Grand Forks ND; ^2^ Centre Nutrition Santé et Société (NUTRISS) Institut sur la Nutrition et les Aliments Fonctionnels (INAF) Université Laval Québec City QC Canada

**Keywords:** genetics, lipidomics, lipids, mass spectrometry, nutrigenomics, Clinical Studies, Lipids and Cholesterol, Metabolism, Biomarkers

## Abstract

**Background:**

Supplementation with long chain n‐3 polyunsaturated fatty acids is used to reduce total circulating triacylglycerol (TAG) concentrations. However, in about 30% of people, supplementation with long chain n‐3 polyunsaturated fatty acids does not result in decreased plasma TAG. Lipidomic analysis may provide insight into this inter‐individual variability.

**Methods:**

Lipidomic analyses using targeted, mass spectrometry were performed on plasma samples obtained from a clinical study in which participants were supplemented with 3 g/day of long chain n‐3 in the form of fish oil capsules over a 6‐week period. TAG species and cholesteryl esters (CE) were quantified for 130 participants pre‐ and post‐supplementation. Participants were segregated into 3 potential responder phenotypes: (1) positive responder (R^pos^; TAG decrease), (2) non‐responder (R^non^; lacking TAG change), and (3) negative responder (R^neg^; TAG increase) representing 67%, 18%, and 15% of the study participants, respectively. Separation of the 3 phenotypes was attributed to differential responses in TAG with 50 to 54 carbons with 1 to 4 desaturations. Elevated TAG with higher carbon number and desaturation were common to all phenotypes following supplementation. Using the TAG responder phenotype for grouping, decreases in total CE and specific CE occurred in the R^pos^ phenotype versus the R^neg^ phenotype with intermediate responses in the R^non^ phenotype. CE 20:5, containing eicosapentaenoic acid (20:5n‐3), was elevated in all phenotypes. A classifier combining lipidomic and genomic features was built to discriminate triacylglycerol response phenotypes and reached a high predictive performance with a balanced accuracy of 75%.

**Conclusions:**

These data identify lipidomic signatures, TAG and CE, associated with long chain n‐3 response p henotypes and identify a novel phenotype based upon CE changes.

**Registration:**

URL: https://www.ClinicalTrials.gov; Unique Identifier: NCT01343342.

**Nonstandard Abbreviations and Acronyms**
CE: cholesteryl ester
DHA: docosahexaenoic acid
EPA: eicosapentaenoic acid
GRS: genetic risk score
LCn‐3: long chain n‐3
R^neg^: negative responder
R^non^: non‐responder
R^pos^: positive responder
sPLSDA: sparse partial least squares discriminant analysis

**Clinical Perspective**

**What Is New?**

- Nearly 30% of people do not respond with lowered triacylglycerol (TAG) concentrations following long chain n‐3 (LCn‐3) polyunsaturated fatty acids supplementation; a multi‐omic approach was taken to link lipidomic and genetic signatures with the different TAG response phenotypes.
- Specific lipidomic signatures were determined for TAG and cholesterol esters from people that respond to LCn‐3 supplementation with decreases in TAG and in people that have increases in TAG; following LCn‐3 supplementation, cholesterol ester concentrations were elevated in 79% of the participants with elevated TAG.
- Lipidomic signatures were integrated with a genetic risk score yielding a predictive analysis with a balanced accuracy of 75%.

**What Are the Clinical Implications?**

- This research provides greater insight into the prevention of heart disease by LCn‐3.
- Our results identify mechanisms underlying why some people may benefit from LCn‐3 intake and why other people may not.
- Our results underscore the importance of multi‐omic approaches for targeted disease prevention.

Supplementation with the long chain n‐3 (LCn‐3) polyunsaturated fatty acids (PUFA) docosahexaenoic acid (DHA, 22:6n‐3) and eicosapentaenoic acid (EPA; 20:5n‐3) reduces plasma triacylglycerol (TAG) concentration.^1^, ^2^, ^3^ Variability in response to LCn‐3 supplementation is well‐documented and our research indicates that about 30% of individuals do not decrease their plasma triacylglycerol in response to LCn‐3 treatment.^4^, ^5^, ^6^, ^7^, ^8^ This inter‐individual variability in response likely underlies controversial findings about the efficacy of LCn‐3 supplementation for prevention of cardiovascular disease (CVD) and has generated efforts to understand the nutrigenetic linkages to LCn‐3 responses.

Advancements in lipidomic technologies allow refined exploration of the relationship of circulated lipids and CVD by providing analysis of specific lipid species. Data from large prospective, clinical studies demonstrate positive and negative associations of targeted lipid species to CVD. For example, lipidomic profiling of plasma samples from participants (n=685) in the Bruneck study identified TAG of 50 to 54 carbons with ≤5 desaturations and the cholesteryl ester (CE) CE 16:1, as directly associated with CVD events.^9^ Similarly, direct correlations of smaller TAG with high saturation and inverse correlations of CE and other lipids with higher number of desaturations with CVD was observed in a cohort of participants (n=983) from the PREDIMED (Prevención con Dieta Mediterránea) study.^10^, ^11^ Lipidomic profiling of participants (n=3779) from the ADVANCE (Action in Diabetes and Vascular Disease: Preterax and Diamicron‐MR Controlled Evaluation) study investigated lipidomic correlates with CVD in people with type 2 diabetes mellitus. Elevated concentrations of saturated and monounsaturated CE, ceramides, and phosphatidylcholines were positively associated with CVD events; whereas TAG 56:6 was negatively associated.^12^

Other than clinical measurements of plasma and serum TAG concentrations, the characterization of which TAG species are modified by LCn‐3 supplementation has not been explored. We do not know whether changes in TAG concentrations are comprehensive in nature or reflect a targeted subset of species. In this work, using samples derived from an LCn‐3 supplementation trial designed to determine nutrigenetic interactions with LCn‐3 responses, we compared the plasma TAG and CE species modified by LCn‐3 supplementation in people who respond with reductions in plasma TAG concentrations versus those people with a neutral response or have increases in plasma TAG concentrations.^13^, ^14^, ^15^, ^16^ Integrating lipidomic data and genomic features allowed for linking changes in specific lipid TAG and CE species to a previously developed genetic risk score^16^ that will assist with predicting response phenotypes.

## Methods

The authors declare that all supporting data are available within the article and its online supplementary files. Concentrations of all lipid species determined are provided in Data S1.

### Clinical Study

The study design has been described in detail previously.^14^ To be eligible, subjects had to be non‐smokers and free of any thyroid or metabolic disorders requiring treatment such as diabetes mellitus, hypertension, severe dyslipidemia, and coronary heart disease. Participants were between ages 18 and 50 years with a body mass index between 25 and 40 kg/m^2^. Subjects were excluded from the study if they had taken n‐3 PUFA supplements in the 6 months before the start of the study. A total of 210 unrelated subjects completed the n‐3 PUFA supplementation period. Two subjects had missing pre‐supplementation values and were excluded from subsequent analyses. The experimental protocol was approved by the ethics committees of Laval University Hospital Research Center and Laval University and participants gave written informed consent. This trial was registered at ClinicalTrials.gov as NCT01343342.

Subjects followed a run‐in period of 2 weeks in which dietary specifications were given about n‐3 PUFA dietary intake: no more than 2 fish or seafood servings per week (maximum of 150 g/week), to choose white‐flesh fishes instead of fatty fishes (examples were given), and to avoid enriched n‐3 PUFA dietary products such as some milks, juices, breads, and eggs. Subjects were also asked to limit their alcohol consumption during the protocol; 2 regular drinks per week were allowed. Subjects were not allowed to take n‐3 PUFA supplements (such as flaxseed), vitamins, or natural health products during the protocol.

After the 2‐week run‐in period, each participant was invited to continue the protocol in compliance with the nutritional recommendations introduced in the run‐in and received a bottle containing the needed n‐3 PUFA capsules for the following 6 weeks. They were invited to take 5 g/day of fish oil (Ocean Nutrition, Nova Scotia, Canada), providing a total of 3 g/day of n‐3 PUFAs (1.9–2.2 g of EPA and 1.1 g of DHA). Compliance was assessed from returned bottles. Subjects were asked to report any deviation during the protocol and to record their alcohol and fish consumption as well as any side effects. Blood samples were collected from an antecubital vein into vacutainer tubes containing EDTA after a 12‐hour overnight fast and 48‐hour alcohol abstinence. Plasma was isolated and frozen at −80°C. Concentrations for glucose, insulin, ApoB, total cholesterol, HDL, and LDL in plasma were those determined previously using routine clinical analyses.^14^ Values for plasma concentrations of triacylglycerol were also determined using a clinical autoanalyzer as described previously.^14^ Samples from 193 participants were shipped to the United States Department of Agriculture Grand Forks Human Nutrition Research Center (Grand Forks, ND, USA) for lipidomic analysis.

### Lipidomic Analysis

Quantitative determination of TAG and CE was performed using multiple internal standards.^17^, ^18^, ^19^ Plasma (20 µL) was combined with 10 µL of internal standard solution in chloroform and an additional 2.0 mL of chloroform. A 150 mg portion of silicic acid was added to the test tube and mixed by vortex for 30 seconds twice with a 10‐minute settling time between mixing to allow adsorption of the aqueous components and phospholipids. For infusion, a 100 µL portion of the sample was transferred to a 350 µL conical insert, dried under argon, reconstituted with mobile phase and capped. Samples were analyzed within 24 hours following previously published methods with modifications to automate sample infusion as detailed in the supporting information.^17^, ^19^

For each sample, 3 separate 50‐µL injections were performed: enhanced mass spectrum analysis from m/z = 770 to 1000 for accurate quantitation of TAG species by brutto‐structure, a neutral loss scan over the same region for 24 common fatty acids to determine relative contributions of each fatty acid to specific brutto‐structures. CE were quantitated using a neutral loss scan for 20 fatty acids from m/z = 400 to 750 with a confirmatory product ion scan for m/z = 369 representing the cholesterol head group. Detailed descriptions of instrument settings and validation have been published elsewhere.^17^ Spectra were processed using LipidView (Framingham, MA, USA) with targeted methods (See Data S1). Isotopic and empirical correction factors for these targets were determined as detailed elsewhere.^17^, ^19^ For CEs, the processing method was modified to include empirically determined ionization correction factors relative to the internals standard as detailed in the Supplementary Methods (Data S2).

### Data Processing and Statistical Analysis

Of the 208 participants who completed the treatment protocol, plasma samples (pre‐supplementation and post‐supplementation) from 193 participants were analyzed by infusion mass spectrometry for TAG and CE.

A percentage change in TAG concentrations was calculated for the clinical chemistry derived values as reported previously^14^ as well as for the sum of the TAG concentrations determined by mass spectrometry using the equation $\%\text{change}=\left(\left(\frac{\left[\text{TAGpost}\right]}{\left[\text{TAGpre}\right]}\right)‐1\right)*100)$. The same equation was used for determining a percentage change in CE following supplementation.

To assure congruity between previously published data^14^ and the results provided herein, we compared the percentage change in sum TAG concentrations determined previously to the percentage change in TAG concentrations as determined by the mass spectrometry method using linear regression analysis (GraphPad Prism version 8.0.0 for Windows, GraphPad Software, San Diego, California USA, www.graphpad.com). This analysis provided a line with slope of 0.83 and r^2^ = 0.77. Residual plots comparing the percentage change determined by both methods of TAG determination were generated and participant percentage change values were used for further analysis if they deviated <±10% from the best fit line. Selection of those values (130 participants) resulted in a linear regression comparison with an r^2^ of 0.93 and a slope of 0.87 (Figure S1).

Lipidomic data, comprised as percentage change in the plasma concentration pre‐supplementation and post‐supplementation for individual TAG and CE species, were analyzed using MetaboAnalyst 4.0 software.^20^ Data were normalized using pareto scaling and compared by one‐way ANOVA with Tukey contrasts. Significance was taken as *P*<0.05 with an applied false discovery rate. Sparse partial least squares discriminant analysis (sPLSDA) was used to determine TAG species that differed between phenotypes.

Differences in anthropometrics, plasma measures of glucose homeostasis and gross plasma lipids were assessed on JMP 15.0.0 (SAS, Cary NC). T‐tests were employed to determine differences within phenotypes between pre‐ and post‐intervention measures. A one‐way ANOVA was used to determine differences between phenotypes in the change from pre‐ to post‐intervention measures with Tukey post hoc analysis. Data were transformed to normal distribution by an optimized Box‐Cox transformation when residual errors were not normally distributed.

For genomic analysis, a principal component analysis was conducted to group TAG species into factors in SAS statistical software v9.4. Factors were kept according to the overall interpretation of eigenvalues, scree plot and cumulative proportion of variance explained by factors. TAG species with a factor loading ≥ 0.5 or ≤−0.5 were kept in respective principal component analysis‐derived factors. Associations between the post‐supplementation factors and a genetic risk score (GRS) were tested in a general linear model adjusted for age, sex, body mass index, and pre‐supplementation factors. GRS development, single nucleotide polymorphism analysis and selection were previously described.^16^ Briefly, the GRS was constructed from mapping refinement of genome‐wide association study signals. Correlations between the percentage change of each TAG and CE species and the GRS were computed and plotted with circlize R package.^21^ From the 130 participants finally included in the present study, genetic data were available for 90 participants to perform genomic analysis.

### Predictive Analysis

A classifier made of lipidomic and genomic features and intended to correctly discriminate TAG response phenotypes was built. The classifier was developed and tested with DIABLO (Data Integration Analysis for Biomarker Discovery Using Latent Components), an integrative method combining data from multiple sources for discriminating between phenotypic groups.^22^ Briefly, DIABLO uses Projection to Latent Structure (PLS), and extends both sPLSDA to multi‐omics analyses and sparse Generalized Canonical Correlation Analysis to a supervised analysis framework.^23^ For predictive purposes, we first reassigned non‐responders as negative responders, and 90 participants were randomly and equally split (45 participants each) into train and test data sets, each containing 17 negative responders and 28 positive responders. Pre‐supplementation plasma levels of those lipid forms whose percentage change was identified as a relevant feature in the sPLSDA were used as input factors for the classifier, as well as coding information from the 31 single nucleotide polymorphisms (Table S1) used to build the aforementioned GRS. The classifier was first tuned and trained in the train data set, and its predictive performance was further tested in the test data set. The performance of the classifier was evaluated by the balanced error rate based on centroid distance.^24^ Predictive analysis with DIABLO is implemented in the mix0mics R package.^24^

## Results

**Figure 1:**
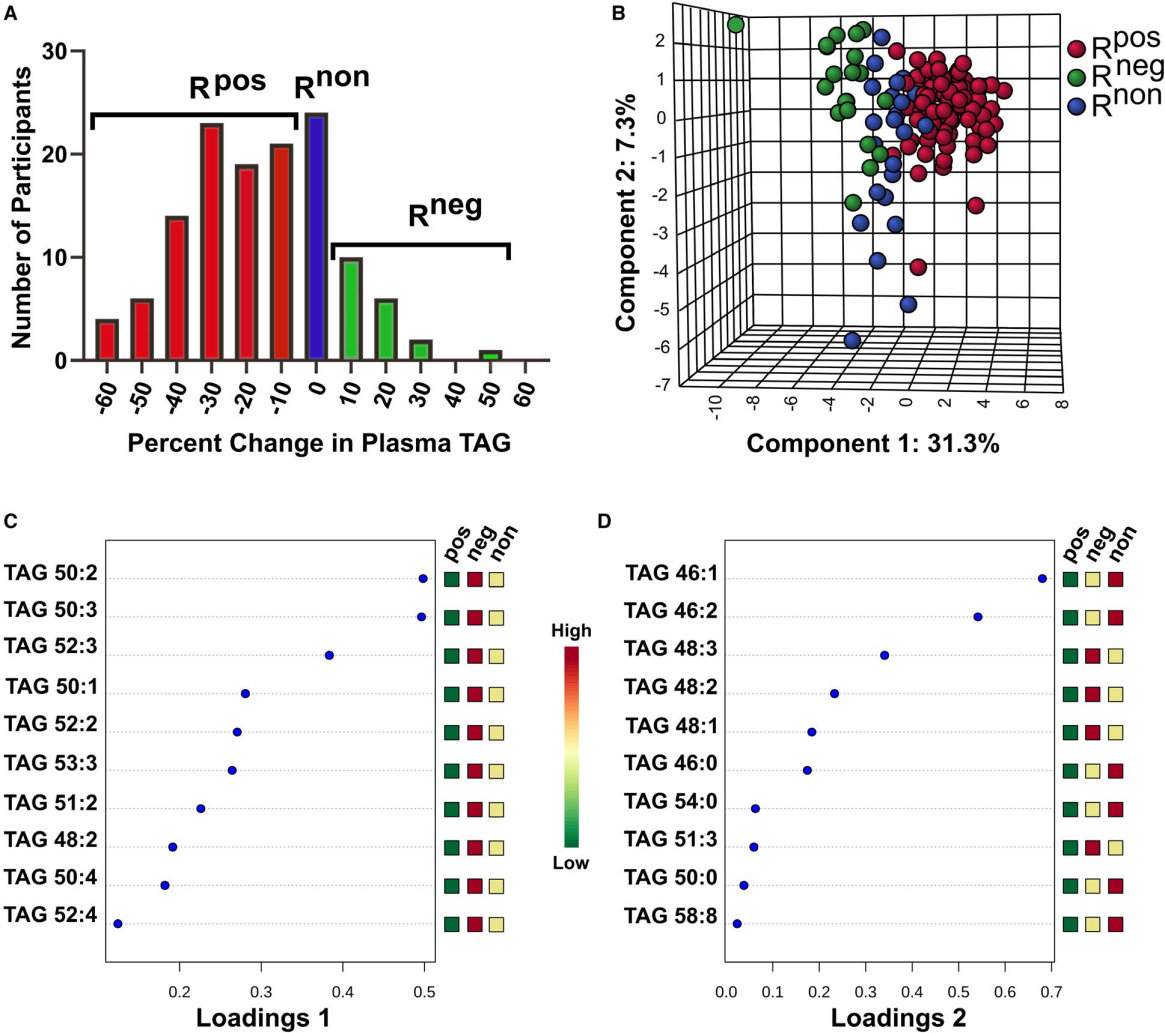
Distribution of triacylglycerol (TAG) response phenotypes and underlying TAG species differences. The distribution of changes in plasma TAG concentrations following LCn‐3 supplementation for participants (**A**). Sparse partial least squares discriminant analysis plot demonstrating separation of response phenotypes (**B**). TAG species and their phenotype groups differences underlying separation along Component 1 of the sparse partial least squares discriminant analysis plot (**C**). TAG species and their phenotype groups differences underlying separation along component 2 of the sparse partial least squares discriminant analysis plot (**D**). A scale bar for relative differences for the Loadings plots is provided. Phenotypes: Positive responder (sum TAG decrease < −10%; n=87), non‐responder (sum TAG changes +/‐ 10%; n=24), and negative responder (sum TAG increase >10%; n=19). R^pos^ indicates positive responder; R^non^, non‐responder; and R^neg^, negative responder.

Participants were sorted into the following phenotypes based upon the percentage change in plasma TAG as determined by mass spectrometry: (1) positive responder (R^pos^; TAG decrease <−10%), (2) non‐responder (R^non^; TAG changes +/‐ 10%), and (3) negative responder (R^neg^; TAG increase >10%). We chose a conservative cut‐off parameter of +/‐ 10% change to account for daily variations in pre‐ or post‐supplementation blood sampling. These phenotypes represented 87/130 (67%), 24/130 (18%), and 19/130 (15%) of the study samples, respectively. The number of participants with the percentage change in 10% increments is shown in Figure 1A. The majority of R^pos^ individuals had between 10% and 40% decreases in TAG concentrations. The majority of R^neg^ individuals had between 10% and 20% increases in TAG concentrations.

Characteristics for the 130 participants distributed into the response phenotypes are presented in Table 1. Fasting plasma glucose was elevated by LCn‐3 supplementation in the R^pos^ and R^non^ phenotypes, but there was not a phenotype‐dependent effect. Insulin concentrations were elevated by LCn‐3 treatment in the R^non^ phenotype. Plasma ApoB concentrations were increased after supplementation in the R^neg^ phenotype. There were phenotype‐dependent changes in plasma TAG concentrations determined by a clinical chemistry analyzer in the previous study and by mass spectrometry in this study.^14^ Decreases in total cholesterol were observed for the R^pos^ phenotype; whereas elevations in total cholesterol occurred in the R^neg^ phenotype. Supplementation resulted in an increase in HDL‐ cholesterol in the R^pos^ phenotype. Baseline characteristics for body mass, body mass index, age, and sex did not differ between phenotypes (Table S2).

**Table 1:** Characteristics of Triacylglycerol Response Phenotypes Following Long Chain n‐3 Supplementation

	R^pos^ (n=87)			R^non^ (n=24)			R^neg^ (n=19)			*P* value Phenotype X pre to post
Characteristics	Pretreatment	Post‐treatment	*P* value (pre‐ to post‐treatment)	Pretreatment	Post‐treatment	*P* value (pre‐ to post‐treatment)	Pretreatment	Post‐treatment	*P* value (pre to post)	
Weight (kg)	80.98 ± 13.3	81.1 ± 13.5	0.41	83.3 ± 18.0	83.7 ± 18.2	0.17	81.1 ± 11.9	81.6 ± 12.2	0.07	0.30
BMI (kg/m^2^)	28.08 ± 3.85	28.12 ± 3.95	0.38	28.0 ± 3.70	28.1 ± 3.80	0.15	27.2 ± 2.70	27.3 ± 2.90	0.07	0.35
Glucose (mmol/L)	4.97 ± 0.48	5.05 ± 0.50	0.03	4.95 ± 0.54	5.22 ± 0.56	0.02	4.95 ± 0.34	5.04 ± 0.44	0.32	0.13
Insulin (pmol/L)	89.2 ± 94.8	79.0 ± 36.4	0.21	91 ± 49.9	109 ± 52.1	<0.01	77.8 ± 46.1	84.7 ± 59.7	0.24	<0.01
ApoB (g/L)	0.87 ± 0.23	0.89 ± 0.21	0.36	0.85 ± 0.28	0.88 ± 0.25	0.30	0.87 ± 0.28	0.96 ± 0.27	<0.01	0.04
Triacylglycerol (mmol/L)										
Clinical analyzer	1.29 ± 0.64	0.89 ± 0.40	<0.01	1.37 ± 0.71	1.37 ± 0.68	0.93	1.08 ± 0.58	1.25 ± 0.69	<0.01	<0.01
Mass spectrometry	1.52 ± 0.74	1.02 ± 0.49	<0.01	1.56 ± 0.82	1.55 ± 0.80	0.99	1.19 ± 0.72	1.44 ± 0.87	0.03	<0.01
ApoB:triacylglycerol	0.78 ± 0.31	1.11 ± 0.37	<0.01	0.70 ± 0.26	0.73 ± 0.27	0.21	0.92 ± 0.33	0.87 ± 0.31	0.63	<0.01
Cholesterol (mmol/L)										
Total	4.85 ± 0.86	4.71 ± 0.88	0.02	4.81 ± 1.04	4.78 ± 0.99	0.78	4.75 ± 1.11	5.01 ± 1.14	0.02	0.01
LDL	2.88 ± 0.75	2.86 ± 0.77	0.67	2.76 ± 0.87	2.76 ± 0.81	0.98	2.94 ± 1.07	3.12 ± 1.09	0.07	0.24
HDL	1.37 ± 0.32	1.44 ± 0.4	<0.01	1.42 ± 0.34	1.4 ± 0.34	0.54	1.31 ± 0.30	1.32 ± 0.32	0.90	0.07
Total cholesterol:HDL	3.68 ± 0.93	3.46 ± 0.97	<0.01	3.59 ± 1.36	3.64 ± 1.35	0.57	3.74 ± 0.99	3.97 ± 1.08	0.02	<0.01

T‐tests were performed between pretreatment and posttreatment values within each phenotype. A single factor ANOVA was performed on pretreatment values but no significant differences were found. Single factor ANOVA was performed on the differences between pretreatment and post‐treatment and Tukey post hoc test was performed to determine the differences between phenotypes. Insulin and triacylglycerol concentrations were not normally distributed and were transformed by the Box‐Cox equation for optimized normal distribution. ApoB indicates apolipoprotein B; BMI, body mass index; HDL, high‐density lipoprotein; LDL, low‐density lipoprotein; R^pos^, positive responder; R^non^, non‐responder; and R^neg^, negative responder.

In order to identify TAG species differing between the 3 phenotypes, sPLSDA was performed. sPLSDA is a supervised data reduction technique commonly used in metabolomics.^25^, ^26^ Figure 1B demonstrates resolution of the responder phenotypes within component 1 of the sPLSDA plot. Component 1 accounts for the majority (31.3 %) of the variance between the phenotypes. The 10 TAG species that were the largest factors, in descending impact, for discriminating the 3 phenotypes (R^pos^, R^neg^, and R^non^) along component 1 are shown in Figure 1C. The TAG species identified mostly have 50 to 53 carbons containing 0 to 3 desaturations. Reductions in these TAG were observed in the R^pos^ phenotype, with no change or increases in the R^non^ and R^neg^ phenotypes, respectively. Separation along component 2 impacted participants mostly in the R^neg^ and R^non^ phenotypes (Figure 1B). The 10 TAG species that were the largest factors impacting separation were TAG with 46 carbons and 48 carbons with 0 to 3 desaturations (Loadings plot 2; Figure 1D). These TAG were reduced in the R^pos^ phenotype; however, R^non^ and R^neg^ phenotypes showed differences in response for 46 carbon versus 48 carbon TAG.

**Figure 2:**
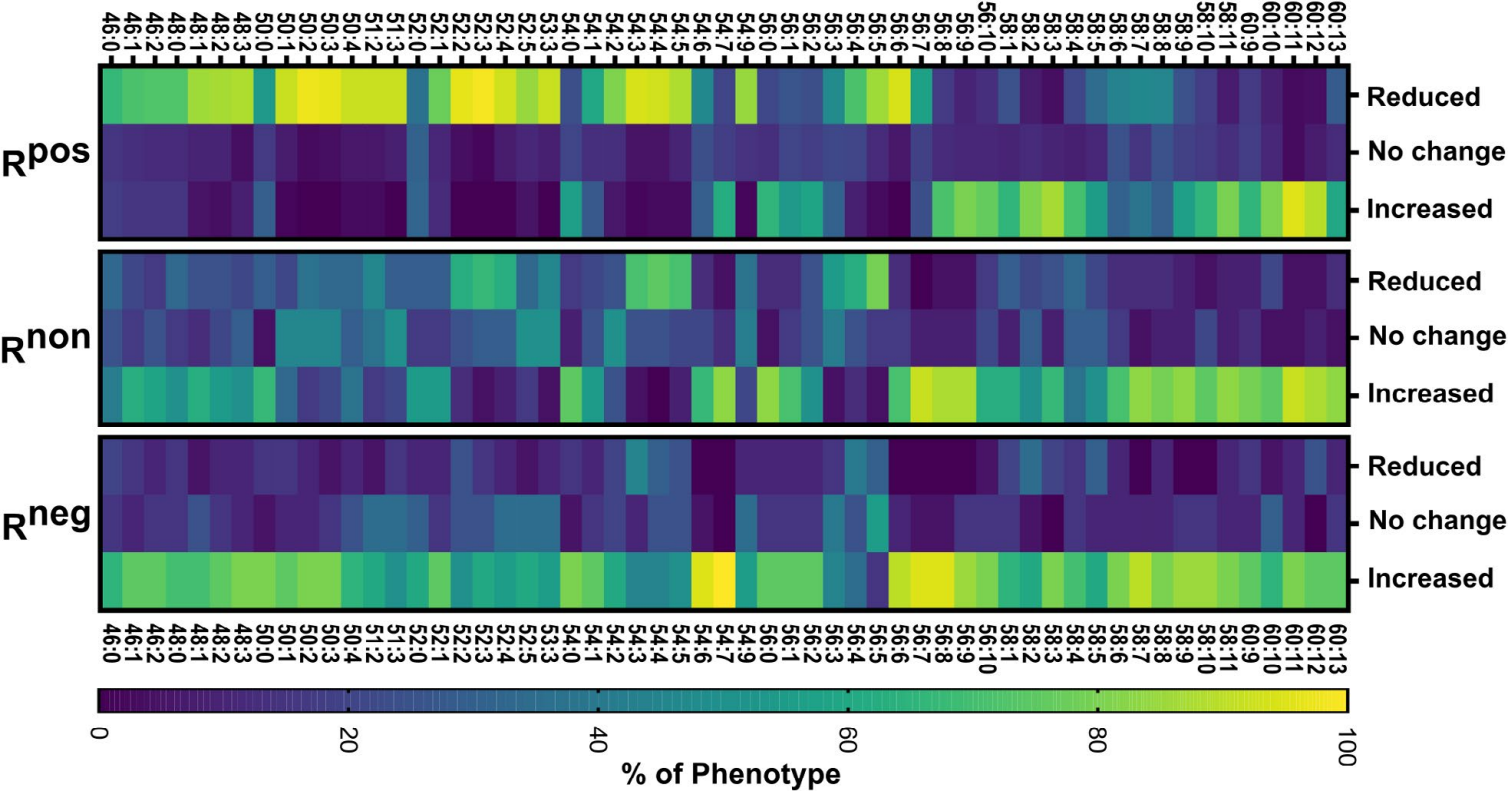
Heatmap analysis of change in triacylglyercol (TAG) species by response phenotype. The percentage of individuals within a given phenotype (y axis) vs the qualitative change in the specific TAG specie (x axis) is shown. A specific TAG was defined as increased (% change>10%), decreased (% change < −10%), or unchanged (changes +/‐ 10%) post‐long chain n‐3 supplementation. The scale bar indicates the percent of a given phenotype that demonstrated a decrease, no change, or increase for a given TAG specie. Phenotypes: Positive responder (sum TAG decrease < −10%; n=87), non‐responder (sum TAG changes +/‐ 10%; n=24), and negative responder (sum TAG increase>10%; n=19). R^pos^ indicates positive responder; R^non^, non‐responder; and R^neg^, negative responder.

To further clarify TAG species indicative of phenotypic identity, we evaluated the degree to which a change in TAG species occurred within a given phenotype designation using the same criteria for phenotypic segregation (Figure 2). As shown in the heatmap, distinct clusters of TAG species comprised TAG 48:1 to 3, to 3, TAG 54:2 to 5, and TAG 56:3 to 6, decreased in most R^pos^ individuals. In contrast to the R^pos^ phenotype, most TAG were elevated in a majority of the R^neg^ phenotype. Of interest, the R^non^ phenotype had characteristics of both the R^neg^, at lower mass TAG, and R^pos^, at the higher mass TAG. We did not observe TAG that remained unchanged between phenotypes.

TAG 56 and TAG58 with ≥7 desaturations and TAG 60 lipids were elevated in all phenotypes and likely represent long chain PUFA‐containing TAG (Figures S2‐S6). TAG58:6 to 8 represented an inflection point in the R^pos^ phenotype. We noted that the highly desaturated TAG 60 species were difficult to evaluate in terms of a percentage increases over baseline because of the fact that these species were only observed in post‐supplementation samples for the most participants. While these TAG are low in concentration, use of neutral loss scanning analysis demonstrates that these species contain EPA and DHA (Figures S3‐S6).

**Figure 3:**
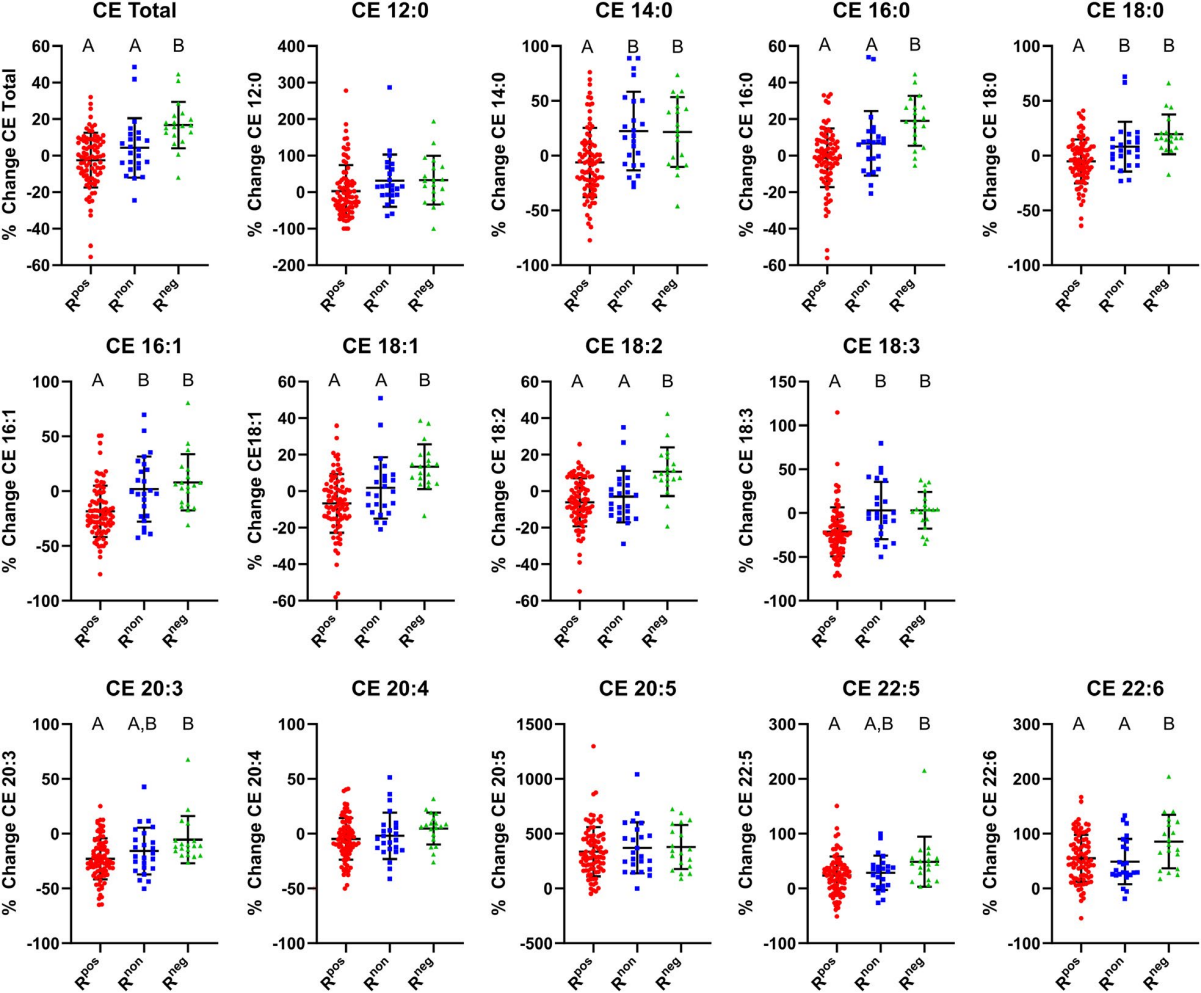
Triacylglcyerol (TAG) response phenotype distinguishes plasma cholesterol ester (CE) responses following LCn‐3 supplementation. Plasma CE responses for total and individual CE were calculated as a percentage change from baseline following long chain n‐3 supplementation. Individual participant data points are provided. Comparisons of phenotypic response for total CE and individual CE were performed by one‐way ANOVA with Tukey post hoc test. Columns without a common superscript letter differ, *P*≤0.05, with applied false discovery rate. Positive responder (n=87), non‐responder (n=24), and negative responder (n=19). R^pos^ indicates positive responder; R^non^, non‐responder; and R^neg^, negative responder.

Given the importance of cholesterol metabolism to CVD risk, we assessed the impact of LCn‐3 PUFA supplementation upon CE based upon TAG phenotype classification (Figure 3, Figure S7). These data demonstrate phenotypic differences in CE responses to supplementation. The mean CE changes for the R^pos^ phenotype were lower than those for the R^neg^ phenotype except for CE 12:0, CE 20:4, and CE 20:5. It is important to note that the R^pos^ phenotype included several individuals for whom decrements in total CE concentrations occurred following treatment. The R^non^ phenotype showed variability in differences with respect to the R^pos^ and R^neg^ phenotypes. Phenotypic differences were not observed for the CE of arachidonic acid (CE 20:4) and eicosapentaenoic acid (CE 20:5). CE 20:5 was elevated in all phenotypes. On the other hand, there existed a greater increase in the elevation of CE 22:6, derived from DHA (22:6n‐3), in the R^neg^ phenotype versus the R^pos^ and R^non^ phenotypes.

CE responses categorized by TAG phenotype are further examined in Table 2. These data demonstrate that 26% of individuals in the R^pos^ phenotype had substantial decreases in CE; whereas 79% of individuals of the R^neg^ had a substantial increase in CE. More than half of the R^pos^ and R^non^ phenotype demonstrated no change in CE concentration following LCn‐3 supplementation.

**Table 2:** Distribution of Cholesterol Ester Changes within Triacylglycerol Response Phenotypes

Phenotype	R^pos^ (n=87)		R^non^ (n=24)			R^neg^ (n=19)	
	Individuals	%	Individuals		%	Individuals	%
Change Total CE*							
Decrease	23	26	4	17		1	5
No change	50	57	14	57		3	16
Increase	14	16	6	26		15	79

* A decrease is defined as a change from baseline < −10%; no change is defined as a change with +/‐ 10% of baseline, and an increase is defined as an increase >10% over baseline. CE indicates cholesteryl ester; R^pos^, positive responder; R^non^, non‐responder; and R^neg^, negative responder.

### Genetic Risk Score and Predictive Analysis

**Figure 4:**
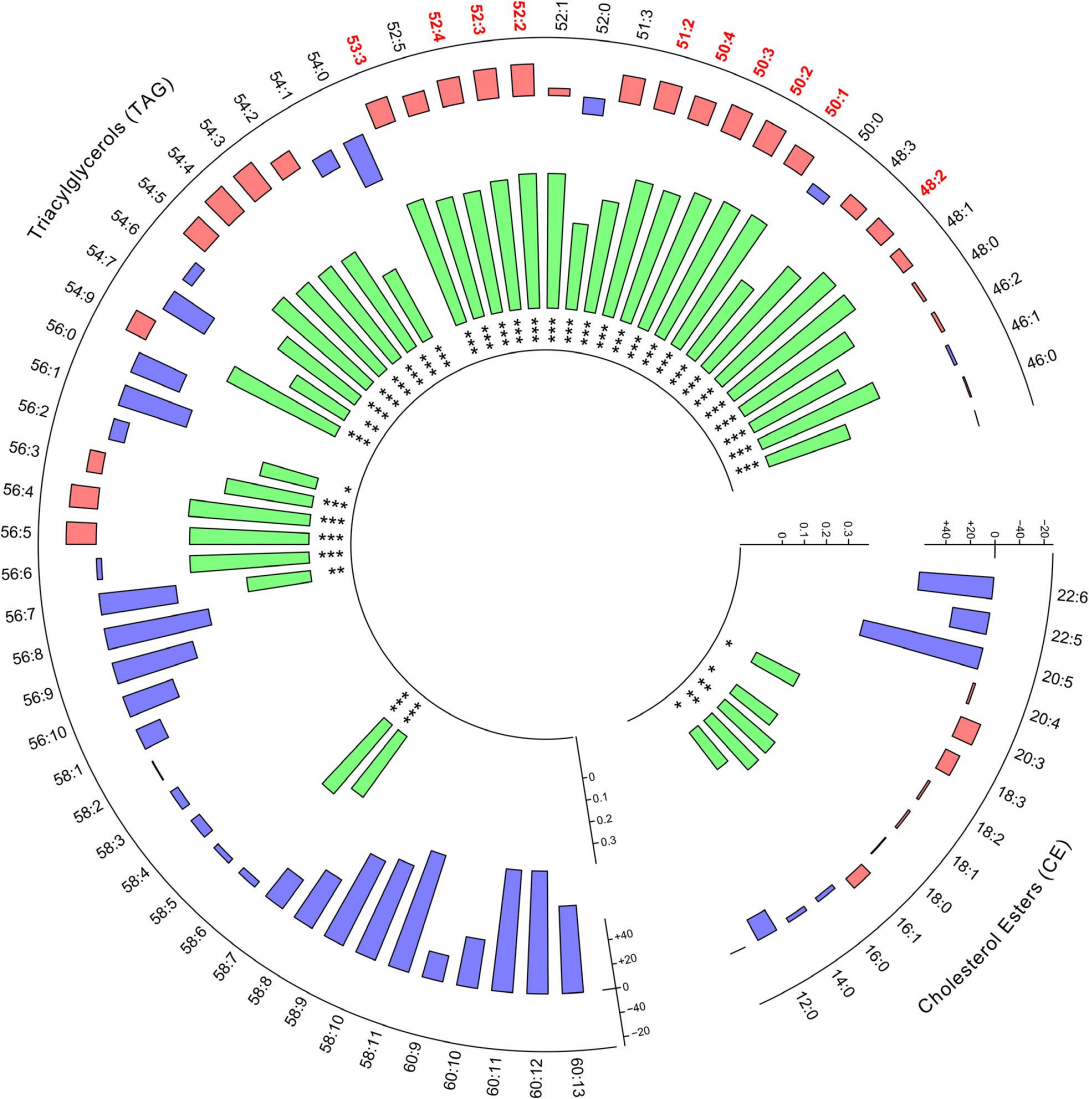
Correlations between percent change of triacylglycerol (TAG) and cholesterol ester (CE) species following long chain n‐3 polyunsaturated fatty acid (LCn‐3) supplementation and the genetic risk score. The percentage change of each TAG and CE species is displayed in the outer layer by the red (percentage decrease) and blue bars (percentage increase). Labels highlighted in red represent those TAG identified as loadings of the factor 1 in the sparse partial least squares discriminant analysis. Significant Pearson correlation coefficients (r) are represented in the inner layer by the green bars. The asterisks represent Pearson correlation *P* values: *P<0.05, **P<0.01, ***P<0.001.

Four factors of TAG species, explaining 82.1% of the variance, were kept following principal component analysis (Table S3). Two of the 4 factors (factor 1 and factor 2), mainly containing short‐chained, highly desaturated TAG, were significantly associated with the GRS, accounting for 17.6% and 21.3% of the variance (*P*<0.0001, for both). Correlations between the percentage change of each TAG and CE species and the GRS are presented in Figure 4. The 10 TAG species previously identified as loadings of factor 1 in the sPLSDA, ie, the most important features for resolving responder phenotypes, were found among the most highly correlated with the GRS and are highlighted in red in Figure 4.

Pre‐supplementation plasma TAG levels of factor 1 loadings were chosen as the lipidomic input data for the classifier. Since the GRS was built to identify TAG response phenotypes based on the TAG percentage change, the global correlation coefficient between the GRS and pre‐supplementation plasma TAG levels was less (r = 0.3) than that observed with TAG percentage changes (r = 0.7). Combined, lipidomic and genomic data reached an overall balanced error rate of 0.26 (26% of misclassification) in the train data set after 10‐fold cross‐validation. The predictive performance of the classifier improved when it was evaluated in the test data set. With a balanced error rate of 0.25, the classifier was able to correctly predict 22 out of 28 positive responders (79%) and 12 out of 17 negative responders (71%).

## Discussion

Given the well‐documented inter‐individual variability in plasma lipid responses following LCn‐3 supplementation, lipidomic characterization of plasma lipids following LCn‐3 supplementation provides insight into the biochemical mechanisms underlying this heterogeneity. In this work, we analyzed lipid responses in samples from a large cohort, nutrigenetic study in which participants were provided LCn‐3 supplementation. By sorting participant responses into R^pos^, R^non^, and R^neg^ phenotypes, we demonstrate clusters of TAG species that decrease following supplementation in the R^pos^ phenotype that are similarly increased in the R^neg^ phenotype. We identified TAG, typically of higher carbon and desaturation number, that were elevated following LCn‐3 supplementation in all phenotypes. A classifier combining lipidomic and genomic features was built to discriminate TAG response phenotypes and reached a high predictive performance with a balanced accuracy of 75%. Lastly, we demonstrate that decreases in plasma CE were observed following supplementation, but that decreases were confined mostly to a subgroup of individuals in the R^pos^, but not R^neg^, phenotype.

In devising the phenotype parameters for R^pos^, R^non^, and R^neg^, we borrowed the pharmacological concepts of an agonist that activates a signaling pathway, an antagonist that blocks activation of the pathway, and an inverse agonist that suppresses the basal activity of a pathway. We segregated individuals who had no net change in plasma TAG concentrations from those who had an increase in plasma TAG concentrations. Our data clearly demonstrate that there are changes in lipid signatures that separate the R^non^ from R^neg^ phenotypes. The R^non^ phenotype is characterized by decreases in some TAG and elevations in others following treatment, unlike the R^neg^ phenotype which demonstrated elevations in most TAG species.

Our data demonstrate that declines in circulating TAG are attributable to reductions in plasma concentrations of TAG species of 48 to 54 carbons with 1 to 4 desaturations. These TAG represent a majority of TAG species present in plasma; a finding observed in people in a smaller feeding trial and in rodents.^17^, ^18^ Based upon the carbon number and desaturations, these TAG consist of the abundant 16 carbon and 18 carbon fatty acids such as palmitate (16:0), oleic acid (18:1n‐9), stearic acid (18:0), linoleic acid (18:2n‐6).

In humans, LCn‐3 have pleiotropic effects that contribute to reduction in circulating TAG concentrations (see the in‐depth review by Shearer et al^2^). These effects include reductions in hepatic production of very low density lipoprotein, clearance of very low density lipoprotein, increases in β‐oxidation and reductions in plasma free fatty acids that are incorporated into very low density lipoprotein.^27^, ^28^, ^29^, ^30^, ^31^ DHA, EPA, and their metabolites may induce these effects by alterations at the transcriptional level via activation of Srebp1, FXR, and PPAR‐mediated mechanisms.^32^, ^33^, ^34^, ^35^ Further research is needed to determine the extent to which these mechanisms are modified in individuals of the R^non^ and R^neg^ phenotypes.

Recent genome‐wide association studies are providing insight into the genes that underpin the differences in LCn‐3 response phenotypes.^14^, ^16^ In the present study, associations between TAG species and a GRS built after fine mapping of genome‐wide association studies hits for plasma TAG response were observed. The GRS was highly associated with 2 of the 4 factors (factors 1 and 2) of TAG species, which were mainly composed of short‐chained TAG species, as opposed to the 2 other factors (factors 3 and 4), for which no association was observed. Consistently, the GRS was mostly correlated with shorter TAG species, whose levels decreased throughout the supplementation as well. These data support the hypothesis that the effect of the GRS is differential depending on TAG length and number of desaturations. Moreover, the predictive performance achieved by the classifier, built with the combination of genomic and lipidomic data, reveals the enormous potential of deep phenotyping in clinical practice. It is worth noting that, even with relatively shallow data, 10 TAG species and 31 single nucleotide polymorphism, and a limited number of participants, we have built an accurate classifier able to correctly identify 8 out of 10 positive responders. Further studies strictly focused on patient classification through multi‐omics signature identification are thus definitely expected in the field.

Our data indicate that phenotypic differences are not resulting from differences in LCn‐3 intake or absorption. CE derived from LCn‐3, in particular CE 20:5, were elevated (>300%) similarly in the R^neg^ phenotype as well as in the R^pos^ and R^non^ phenotypes. The greater increases in CE 22:6 in the R^neg^ phenotype (85%) versus the R^pos^ (55%) or R^non^ (49%) may be the result of differential metabolism of CE containing EPA versus CE containing DHA in the R^neg^ phenotype. LCn‐3 treatment across phenotypes elevated most TAG species of ≥54 carbons and with ≥6 desaturations except for TAG 58:6 in the R^pos^ phenotype. The higher molecular weight, highly desaturated TAG are indicative of incorporation of LCn‐3 into circulation and have been observed previously.^36^

Lipidomic assessment of LCn‐3 incorporation into CE and TAG has been reported previously.^17^, ^37^ Previous research from our laboratory, comprising analysis of plasma from healthy adult participants consuming graded amounts of Atlantic salmon (90 g and 180 g biweekly) as a dietary source of mixed LC‐n3 (EPA, DHA, and docosapentaenoic acid [22:5n‐3]) demonstrated that, similar to our current findings, the CE pool of EPA has a greater capacity for expansion than the CE pool of DHA.^17^ In a study by Pastor and colleagues, lipidomic assessment was performed in children with cystic fibrosis following treatment with a DHA‐containing seaweed oil.^37^ This analysis demonstrated that CE is a major pool for DHA and EPA in the plasma. However, while concentrations of CE 20:5 are comparable, the concentrations of CE 22:6 reported by Pastor and colleagues are ≈5‐fold greater and demonstrate a larger capacity for enlargement compared with our current data (Data S1). We speculate that these differences in the LCn‐3 content of the CE pool may be the result of differences in age and/or health status. Similar to the data by Pastor and colleagues, TAG species represent only a limited pool for LCn‐3 incorporation.

sPLSDA further demonstrated separations between the R^non^ and R^neg^ phenotypes based upon carbon number, lauric acid (12:0), and myristic acid (14:0) are likely fatty acid constituents of the 46 carbon and 48 carbon TAG identified in component 2 of the sPLSDA plot. Neutral loss scan analysis demonstrated that myristic acid and to a lesser extent lauric acid existed in these TAG species (Figures S8 and S9). Given that myristic acid is observed in dairy products, we examined the diet records of the participants. However, no differences in dairy intake were found between phenotypes (data not shown). The underpinnings for the differences in these smaller mass TAG are not clear. We speculate that elevations in these TAG represent an enhanced incorporation of 12:0 and 14:0 derived from de novo lipogenesis into very low‐density lipoprotein.

Concerns with LCn‐3 supplementation exist because of increases in circulating cholesterol concentrations following LCn‐3 supplementation that can occur in some individuals.^5^, ^38^, ^39^, ^40^, ^41^ Our results assist with clarifying this issue. CE concentrations did not change in response to supplementation for nearly half of the participants, but our data show disparities that segregate according to TAG response phenotype. A decline in CE concentrations occurred in a minority of the R^pos^ and R^non^ phenotypes; whereas, increases in CE concentrations occurred in 79% of the R^neg^ individuals. The increase in circulating CE may be a response to a general increase in the export of lipids and lipoproteins into the circulation, an explanation consistent with the elevated concentrations of ApoB particles and TAG in the R^neg^ phenotype. Our data are limited in that we do not know in which lipoprotein compartments these changes in CE occurred.

Our data dovetail with existing lipidomic studies examining the relationship of specific lipidomic signatures to CVD events. Our data demonstrate that reductions in TAG ≤ 54 carbons, with < 5 desaturation occur in R^pos^ individuals. Elevated concentrations of these TAG were directly associated with CVD events in the large cohort Bruneck and PREDIMED studies.^9^, ^10^ Similarly, elevated concentrations of saturated and monounsaturated CE were directly associated with CVD events in the Bruneck and ADVANCE studies.^9^, ^12^ Moreover, lipidomic analysis of human atherosclerotic plaques by Stegemann and colleagues revealed that CE compose approximately half of plaque lipid with CE 18:1 and CE 18:2 as the most abundant CE species.^42^ Our data demonstrate that these CE are reduced to a greater extent in the R^pos^ phenotype versus the R^neg^ phenotype although variability existed in R^non^ responses. We note, however, that within the R^pos^ and R^non^ phenotypes, some individuals still exhibited elevations in these CE. Nonetheless, a proportion of the R^pos^ phenotype displayed reductions in TAG and CE species directly associated with CVD events and may represent individuals for whom LCn‐3 supplementation is beneficial. Importantly, R^neg^ phenotype individuals responded to LCn‐3 supplementation with increases in TAG and CE associated with elevated CVD. A greater understanding of the mechanisms underlying these negative responses to LCn‐3 treatment may have clinical benefit. It is not clear whether individuals of the R^non^ phenotype may benefit from LCn‐3 supplementation given the variable responses in reductions in TAG 52:2 to 4, TAG 54:3 to 5, TAG 56:3 to 5 and the increases in smaller 46 and 48 carbon TAG.

### Limitations and Future Directions

The plasma samples analyzed in this study were from a previously completed clinical study, and there are always concerns about the stability of fatty acids during long‐term storage. Plasma samples were stored at −80°C following collection to limit loss of fatty acids, particularly PUFA, during storage.^43^, ^44^, ^45^, ^46^

Participants in this study received LCn‐3 in the form of fish oil. DHA and EPA have differential effects on plasma lipids.^38^, ^47^ The extent to which supplementation with EPA versus DHA may have differential impact on TAG structures is not known. We acknowledge that this study focused on changes in TAG and CE. While previous analyses demonstrate no phenotypic differences in elevating plasma phospholipid fatty acid content of LCn‐3 as a result of fish oil supplementation, there may be differences in the speciation of the phospholipids and other members of the plasma lipidome including ceramides and oxylipins.^14^, ^48^ Our study speciates TAG only to the level of brutto‐structure, acyl carbon number and desaturations. Subsequent analyses using liquid chromatography‐based separations are needed to further refine the fatty acid composition and position isomers for TAG species. Existing data indicate that genotypic differences associated with difference in LCn‐3 supplementation responses may vary with population.^14^ The degree to which variations in genotypic or epigenetic backgrounds between populations may still yield similar changes in lipidomic signatures is an important, remaining question. There is a need to extend similar lipidomic characterization to other LCn‐3 supplementation studies to determine the extent to which lipidomic changes are related LCn‐3 based reductions in CVD events.

## Conclusions

Research is overwhelmingly pointing to the need of integrated “‐omic” strategies to prevent disease and design therapeutic approaches. In this research, we have refined the understanding of the lipidomic changes that result from LCn‐3 supplementation and have identified TAG and CE species that display phenotypic variability. Our work highlights the existing need for further clarification of the role of specific lipids in the development of CVD and highlights the need for integrating genomic and metabolomic platforms for the prevention of CVD.

## Sources of Funding

This work was supported by US Department of Agriculture, Agricultural Research Service project 3062‐53000‐001‐00D (M.J.P., M.R.B., B.R.), US Department of Agriculture, National Institute of Food and Agriculture award number 2014‐67017‐21758 (M.J.P.). B.V.M. received a scholarship from the Fonds de Recherche du Québec – Santé. M.C.V. is Tier 1 Canada Research Chair in Genomics Applied to Nutrition and Metabolic Health. This work was supported by the Réseau de Recherche en Santé Cardiométabolique, Diabète et Obésité, and the Canadian Institutes of Health Research ‐ (MOP‐110975).

## Disclosures

None.

## Supporting information


**Data S1–S2**

**Tables S1–S3**
**Figures S1–S9**
