# Supplementary material for: Identification of Phenotypic Lipidomic Signatures in Response to Long Chain n‐3 Polyunsaturated Fatty Acid Supplementation in Humans
Source: J Am Heart Assoc. 2021 Jan 19;10(3):e018126. doi: 10.1161/JAHA.120.018126 (PMC7955441; doi:10.1161/JAHA.120.018126)

Supplemental Material

Data S2.

Supplemental Methods

Lipidomic analysis

HPLC-grade chloroform, LC-MS grade ammonium acetate, and butylated hydroxy toluene (BHT) were obtained from Sigma-Aldrich (St. Louis, MO, USA). LC-MS-grade methanol was purchased from Fisher Scientific (Fairlawn, NJ, USA). Triacylglycerol and cholesteryl ester standards were purchased from Nu-Chek Prep. (Elysian, MN, USA) and used as received. UNISIL silicic acid was purchase from Clarkson Chromatography Products (South Williamsport, PA, USA).

Sample Preparation. Plasma samples were thawed on ice and vortexed to ensure homogeneity. A 20 µL aliquot of plasma was combined with 10 µL of internal standard solution in chloroform (471 µM triheptadecanoin, 626 µM cholesteryl heptadecanoate) in a 15 x 100 screw-top test tube. After an additional 2.0 mL of chloroform (100 µM BHT) was added, the sample was vortexed again. At this point the aqueous portion of the plasma floated at the surface of the chloroform. Using a Redding 10X pistol & small rifle powder measure (Cortland, NY, USA), 150 mg of silicic acid was added to the test tube. After the addition of the silicic acid the sample was sealed with Teflon-lined cap and mixed by vortex for 30 seconds. The solid phase settled for ten minutes before repeating the mixing step. At this stage samples consisted a supernatant with the aqueous component of plasma adsorbed to the stationary phase. Samples proved stable for three months when stored at -20 °C in this format. For infusion, a 100 µL portion of the sample was transferred to a 350 µL conical insert (Wheaton MicroLiter,

Millville, NJ, USA), dried under argon and reconstituted with 1:1 methanol: chloroform (10 mM ammonium acetate) and capped with a Teflon-lined septum. At this stage samples were analyzed within 24 hours.

Sample Analysis. Samples were analyzed after preparation following previously published methods with modifications to automate sample infusion [17,19.]. The autosampler and two solvent pumps from a Shimadzu Prominence LC-20XR HPLC system (Shimadzu North America, Columbia, MD, USA) were used to infuse samples into the electrospray source of an AB Sciex 5500 QTRAP hybrid mass spectrometer (Framingham, MA, USA). Pump A delivered 1:1 methanol: chloroform (10 mM ammonium acetate) at a rate of 10 µL/min, pump B delivered buffer-free 1:1 methanol: chloroform at a rate of 50 µL/min between samples in order to clean the line and source. All connections were made with either stainless steel or 100-micron Peeksil tubing. A 0.2 micron stainless steel frit was placed in-line between the autosampler and the electrospray source to capture possible silicic acid carry-over and to provide sufficient back-pressure to ensure stable pump operation.

For each sample three separate 50 µL injections were performed. First an enhanced mass spectrum (EMS) analysis from m/z =770-1000 for accurate quantitation of TAG species by brutto-structure as the ammoniated [TAG C:N + NH_4]+ ion, where C represents the acyl carbon number and N represents the total desaturation level of the acyl chains. This was followed by a neutral loss scan (NLS) over the same region for fatty acid neutral losses in Table S1 to determine relative contributions of each fatty acid to specific brutto-structures. Cholesteryl esters were quantitated using an NLS as

indicated in table S1 from m/z = 400-750 with an additional product ion scan for m/z= 369 representing the cholesterol head group. Detailed descriptions of instrument settings and validation have been published elsewhere [17].

Spectra Processing. Spectra were processed using LipidView (Framingham, MA, USA) with targeted methods for species shown in tables S2 and S3. The derivation of the composite correction factors, which correct for isotopic effects and the differential ionization of lipid species is detailed elsewhere [17]. For cholesteryl esters the processing method was modified to include empirically determined ionization correction factors relative to the internal standard CE 17:0 for CE 12:0, CE: 14:0, CE 16:1, CE 16:0, CE 18:2, CE 18:1, CE 20:5, CE 20:4 and CE 24:1. Values for CE 15:0 and CE 18:0 were interpolated from a least-squares fit of the response factors for the measured saturated species. In cases where either a commercial standard was not available or insufficient data was available for interpolation, values were assigned based upon species with the same acyl carbon number and the nearest desaturation value.

Table S1. Neutral loss scan (NLS) fatty acid species. Spectra for neutral losses marked with “Y” were used for the designated lipid class.

Fatty Acid	Neutral Loss $[FA + NH_4]^0$	TAG	CE
C 12:0	217	Y	Y
C 14:0	245	Y	Y
C 16:1	271	Y	Y
C 16:0	273	Y	Y
C 17:1	285	Y	N
C 17:0	287	Y	Y
C 18:3	295	Y	Y
C 18:2	297	Y	Y
C 18:1	299	Y	Y
C 18:0	301	Y	Y
C 19:0	315	Y	N
C 20:5	319	Y	Y
C 20:4	321	Y	Y
C 20:3	323	Y	Y
C 20:2	325	Y	N
C 20:1	327	Y	N
C 20:0	329	Y	N
C 22:6	345	Y	Y
C 22:5	347	Y	Y
C 22:0	357	Y	N
C 24:1	383	Y	N
C 24:0	385	Y	N

TAG, triacylglycerol; CE, cholesterol ester

Table S2. Enhanced mass spectrum (EMS) of triacylglycerol (TAG) species with composite correction factors.

TAG Species	m/z	Composite Correction Factor	TAG Species	m/z	Composite Correction Factor
TAG 46:2	792.7	1.3966	TAG 56:10	916.6	1.1619
TAG 46:1	794.6	1.6506	TAG 56:9	918.6	1.1960
TAG 46:0	796.6	2.0187	TAG 56:8	920.6	1.2426
TAG 48:3	818.6	1.3129	TAG 56:7	922.6	1.3033
TAG 48:2	820.6	1.5165	TAG 56:6	924.6	1.3826
TAG 48:1	822.6	1.7959	TAG 56:5	926.6	1.4873
TAG 48:0	824.7	2.1997	TAG 56:4	928.6	1.6252
TAG 50:4	844.6	1.2351	TAG 56:3	930.6	1.8134
TAG 50:3	846.6	1.4234	TAG 56:2	932.6	2.0774
TAG 50:2	848.7	1.6357	TAG 56:1	934.7	2.4706
TAG 50:1	850.7	1.9533	TAG 56:0	936.6	3.1217
TAG 50:0	852.7	2.3988	TAG 58:11	942.6	1.2257
TAG 51:3	860.6	1.4816	TAG 58:10	944.6	1.2490
TAG 51:2	862.7	1.7154	TAG 58:9	946.6	1.2837
TAG 52:5	870.6	1.2377	TAG 58:8	948.6	1.3316
TAG 52:4	872.6	1.3817	TAG 58:7	950.6	1.3965
TAG 52:3	874.6	1.5420	TAG 58:6	952.6	1.4845
TAG 52:2	876.6	1.8015	TAG 58:5	954.6	1.5938
TAG 52:1	878.7	2.1244	TAG 58:4	956.6	1.7433
TAG 52:0	880.6	2.6178	TAG 58:3	958.7	1.9478
TAG 53:3	888.6	1.6040	TAG 58:2	960.7	2.2396
TAG 54:9	890.6	1.1148	TAG 58:1	962.6	2.6778
TAG 54:7	894.6	1.2028	TAG 60:13	966.6	1.5079
TAG 54:6	896.6	1.2718	TAG 60:12	968.6	1.6417
TAG 54:5	898.6	1.3633	TAG 60:11	970.6	1.7972
TAG 54:4	900.6	1.4866	TAG 60:10	972.6	2.0146
TAG 54:3	902.6	1.6685	TAG 60:9	974.6	2.3232
TAG 54:2	904.6	1.8990			
TAG 54:1	906.6	2.2642			
TAG 54:0	908.6	2.8696			

Table S3. Cholesteryl ester (CE) species with composite correction factors.

CE Species	m/z	Neutral loss	Composite response factor
CE 12:0	586.7	217	1.743
CE 14:0	614.8	245	1.689
CE 15:0	628.9	259	1.660
CE 16:1	640.9	271	1.049
CE 16:0	642.9	273	1.595
CE 18:3	664.9	295	0.777
CE 18:2	666.9	297	0.777
CE 18:1	668.9	299	0.953
CE 18:0	670.9	301	1.595
CE 20:5	688.8	319	0.530
CE 20:4	690.9	321	0.538
CE 20:3	692.9	323	0.538
CE 20:2	694.9	325	0.538
CE 20:1	696.9	327	0.538
CE 22:5	716.9	347	0.471
CE 22:6	714.8	345	0.471

Table S1. Description of the 31 single nucleotide polymorphisms (SNP) comprising the genetic risk score.

SNP (rs number)	Location	Position (base pairs)[a]	GWAS locus
rs7639707	Intron	159148087	*IQCJ-SCHIP1*
rs62270407	Intron	159597626	*IQCJ-SCHIP1*
rs61569932	Upstream *NXPH1*, intron of *ICA1*	8299207	*NXPH1*
rs1990554	Upstream *NXPH1*, intron of *ICA1*	8344530	*NXPH1*
rs6463808	Intron	8476787	*NXPH1*
rs6966968	Downstream *NXPH1*, intergenic	8840378	*NXPH1*
rs28473103	Downstream *NXPH1*, intergenic	8842073	*NXPH1*
rs28673635	Downstream *NXPH1*, intergenic	8855531	*NXPH1*
rs12702829	Downstream *NXPH1*, intergenic	9049555	*NXPH1*
rs78943417	Downstream *NXPH1*, intergenic	9062499	*NXPH1*
rs293180	Downstream *NXPH1*, intergenic	9159909	*NXPH1*
rs1837523	Downstream *NXPH1*, intergenic	9201284	*NXPH1*
rs1216346	Upstream *PHF17*, intergenic	129555929	*PHF17*
rs114348423	Downstream *PHF17*, intergenic	130112033	*PHF17*
rs75007521	Downstream *PHF17*, intergenic	130286406	*PHF17*
rs72560788	Upstream *MYB*, intergenic	135200886	*MYB*
rs72974149	Upstream *MYB*, intergenic	135395122	*MYB*
rs210962	Intron	135503785	*MYB*
rs6933462	Downstream *MYB*, intergenic	135584967	*MYB*
rs79624996	Upstream *NELL1*, intergenic	20211262	*NELL1*
rs1850875	Intron	20731343	*NELL1*
rs78786240	Intron	20735026	*NELL1*
rs117114492	Intron	21008313	*NELL1*
rs184945470	Upstream *SLIT2*, intergenic	19334808	*SLIT2*
rs143662727	Upstream *SLIT2*, intergenic	19634162	*SLIT2*
rs10009109	Upstream *SLIT2*, intergenic	19655475	*SLIT2*
rs10009535	Upstream *SLIT2*, intergenic	19747014	*SLIT2*
rs61790364	Upstream *SLIT2*, intergenic	19921757	*SLIT2*
rs73241936	Upstream *SLIT2*, intergenic	20008049	*SLIT2*
rs16869663	Intron	20485683	*SLIT2*
rs76015249	Downstream *SLIT2*, intron of *KCNIP4*	20735742	*SLIT2*

[a] Human Genome Assembly GRCh37/hg19.
GWAS: Genome-wide association study.

Table S2. Baseline characteristics of R^{pos}, R^{non}, and R^{neg} phenotypes. A one way ANOVA was performed on continuous variables: body mass, age and BMI*. A Chi-squared analysis was performed on sex.

	R^{pos} (n = 87)			R^{non} (n = 24)			R^{neg} (n = 19)			One way ANOVA P value
Body mass (kg)	81.0	±	13.3	83.3	±	18.0	81.1	±	11.9	0.76
BMI (kg/m^2)	28.1	±	3.8	28.0	±	3.7	27.2	±	2.7	0.61
Age (y)	31.3	±	8.3	29.5	±	8.2	34.9	±	8.6	0.10
Sex	M		F	M		F	M		F	X^2 p value
n	37		50	11		13	10		9	0.72

*BMI, Body mass index; F, female; aM, male; R^{pos}, positive responder; R^{non}, non-responder; R^{neg}, negative responder

Table S3. Composition of triacylglycerol (TAG) factors from principal component analysis.

Triglyceride	Factor 1	Factor 2	Factor 3	Factor 4
TAG 54:4	**0.96**	0.18	0.05	0.02
TAG 54:3	**0.91**	0.27	-0.04	0.11
TAG 54:5	**0.90**	0.27	0.22	-0.03
TAG 56:4	**0.89**	0.30	0.05	0.18
TAG 56:5	**0.84**	0.39	0.17	-0.03
TAG 52:4	**0.84**	0.40	0.21	-0.14
TAG 52:3	**0.84**	0.49	0.10	-0.08
TAG 53:3	**0.82**	0.47	0.10	0.03
TAG 56:3	**0.80**	0.31	0.14	0.31
TAG 52:5	**0.75**	0.47	0.32	-0.11
TAG 54:2	**0.74**	**0.52**	0.03	0.21
TAG 52:2	**0.73**	**0.61**	-0.01	0
TAG 54:9	**0.73**	**0.54**	0.13	0.11
TAG 51:3	**0.70**	**0.55**	0.11	0.03
TAG 56:6	**0.69**	0.42	**0.53**	-0.01
TAG 54:6	**0.66**	0.35	**0.59**	-0.02
TAG 56:2	**0.55**	0.41	0.36	0.44
TAG 58:6	**0.55**	0.44	0.46	0.28
TAG 46:1	0.23	**0.93**	0.16	0.02
TAG 48:1	0.35	**0.91**	0.11	-0.02
TAG 48:0	0.28	**0.89**	0.11	0.10
TAG 48:2	0.42	**0.88**	0.13	-0.06
TAG 46:2	0.26	**0.87**	0.20	0.01
TAG 50:1	0.49	**0.82**	0.05	0.02
TAG 48:3	0.46	**0.82**	0.18	-0.04
TAG 50:2	**0.56**	**0.80**	0.05	-0.06
TAG 52:1	0.42	**0.77**	0.05	0.09
TAG 46:0	0.27	**0.77**	0.14	0.17
TAG 50:3	**0.63**	**0.73**	0.11	-0.11
TAG 51:2	**0.64**	**0.72**	0.03	0.02
TAG 50:0	0.15	**0.69**	0.11	0.31
TAG 50:4	**0.64**	**0.68**	0.21	-0.10
TAG 52:0	0.30	0.44	0.26	0.27
TAG 58:9	0.21	0.09	**0.95**	0.08
TAG 58:10	0.11	0.15	**0.94**	0.12
TAG 56:8	0.24	0.18	**0.94**	0.03
TAG 56:9	0.27	0.19	**0.91**	0.12
TAG 58:11	-0.09	0	**0.88**	0.25
TAG 58:8	0.34	0.10	**0.86**	0.16

TAG 56:7	0.35	0.30	**0.86**	0.01
TAG 60:12	-0.28	-0.03	**0.85**	0.23
TAG 60:11	-0.12	-0.01	**0.83**	0.24
TAG 54:7	0.39	0.33	**0.82**	0.01
TAG 58:7	0.47	0.36	**0.73**	0.11
TAG 56:10	0.35	0.24	**0.69**	0.31
TAG 60:13	-0.27	-0.20	**0.66**	0.43
TAG 60:10	0.06	0.03	**0.66**	0.18
TAG 60:9	0.05	0.09	**0.63**	0.39
TAG 56:0	0.01	0	**0.60**	0.24
TAG 58:2	0.04	0	0.24	**0.82**
TAG 58:1	-0.19	-0.06	0.32	**0.77**
TAG 58:3	0.11	0.12	0.34	**0.61**
TAG 58:4	0.35	0.13	0.31	**0.60**
TAG 56:1	-0.03	0.06	0.48	0.48
TAG 54:1	0.35	**0.50**	0.17	0.26
TAG 54:0	0.17	0.37	0.48	0.10
TAG 58:5	0.25	-0.03	0.37	0.43

Factor loadings ≥ 0.5 or ≤ -0.5 are marked in bold.

Figure S1. Comparisons of percent change in triacylglycerol (TAG) concentrations following long chain n-3 polyunsaturated fatty acid supplementation using a clinical analyzer and mass spectrometry (MS). (A) Linear regression analysis was performed on the entire data set (193 participants) with its residual plot shown in (B). (C) Linear regression analysis for participant data (130 participants) falling within the 10 percent variance highlighted in (B). The resulting residuals plot for the linear regression comparing the percent change for the 130 [illegible]

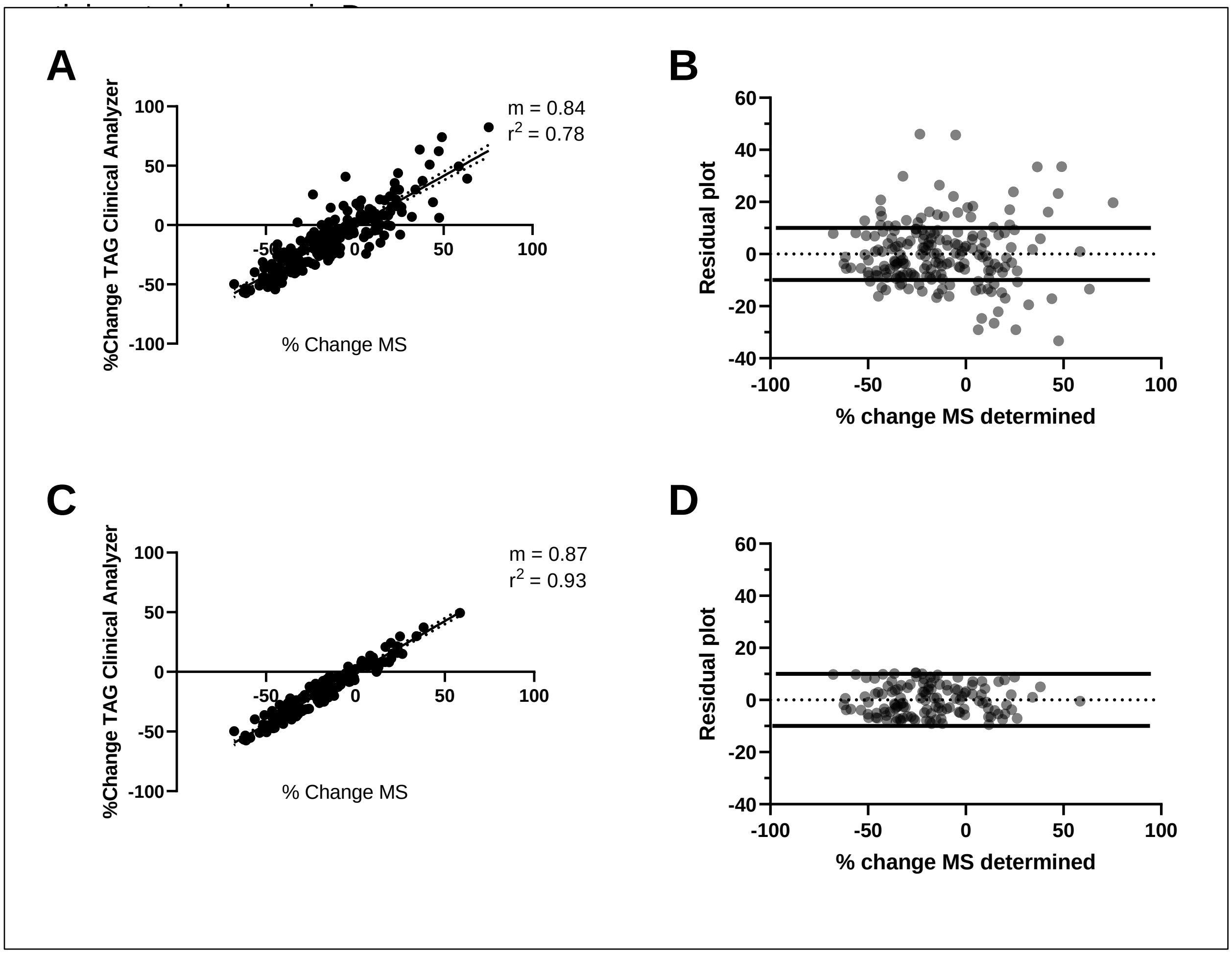

Figure S2. Distribution of triacylglycerol (TAG) species composition in human plasma. Following quantitative determination of TAG species concentration by brutto structure, TAG species were subsequently expressed as a percent of the total TAG concentration. TAG 52:2-4 comprise 43.4% of total TAG in human plasma. TAG 50:1-3 and TAG 54:3-5 comprise over 14% of TAG, respectively. Note that many species comprise less than 1% of the total TAG concentration. Data presented are for the R^{pos} phenotype (n = 87). Data are the mean ± SD.

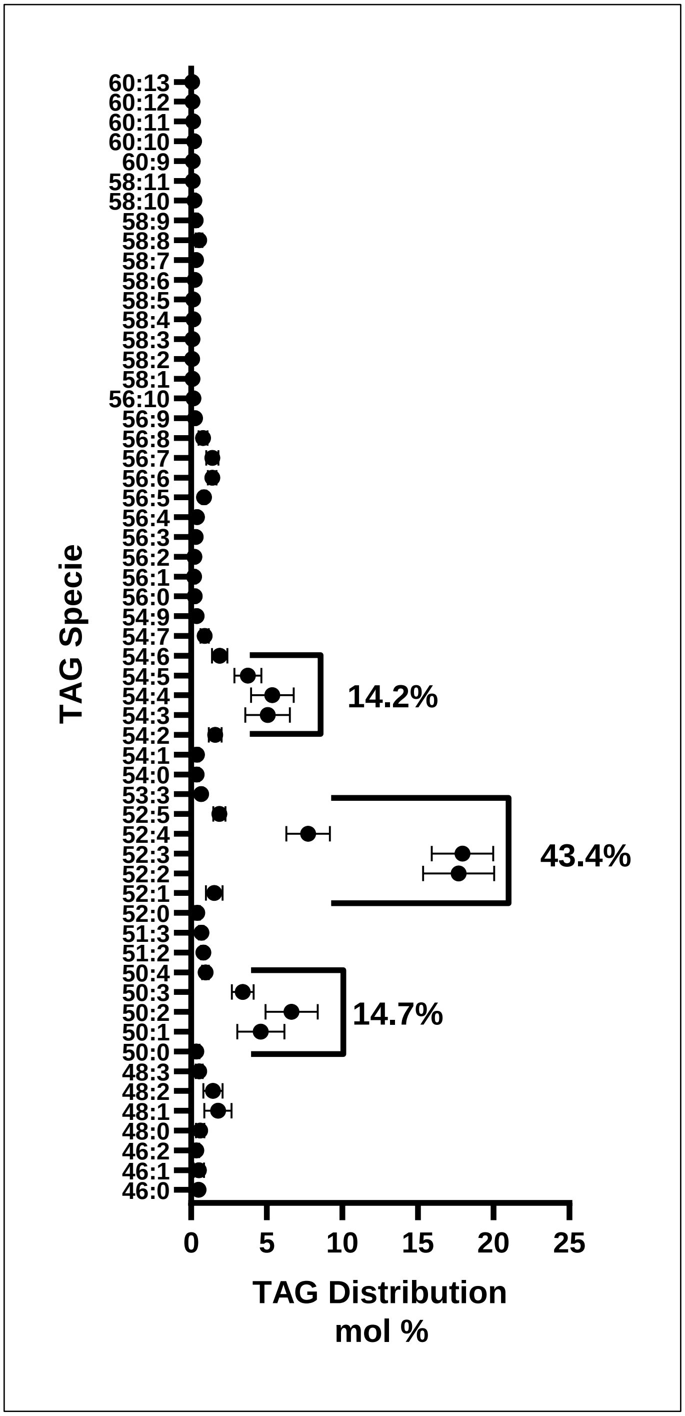

Figure S3. Characterization of incorporation of long chain polyunsaturated fatty acids (LCPUFA) into plasma triacylglycerol (TAG) species. Neutral loss scans (NLS) for arachidonic acid (fatty acid (FA) 20:4) and eicosapentaenoic acid (FA 20:5) EPA and pre- and post- treatment for a R^{neg} phenotype individual. The spectrum is limited to the region where $[TAG\ 56{:}X + NH_4]^+$ and $[TAG\ 58{:}X + NH_4]^+$ appear, where X is the desaturation level at the top of each peak.

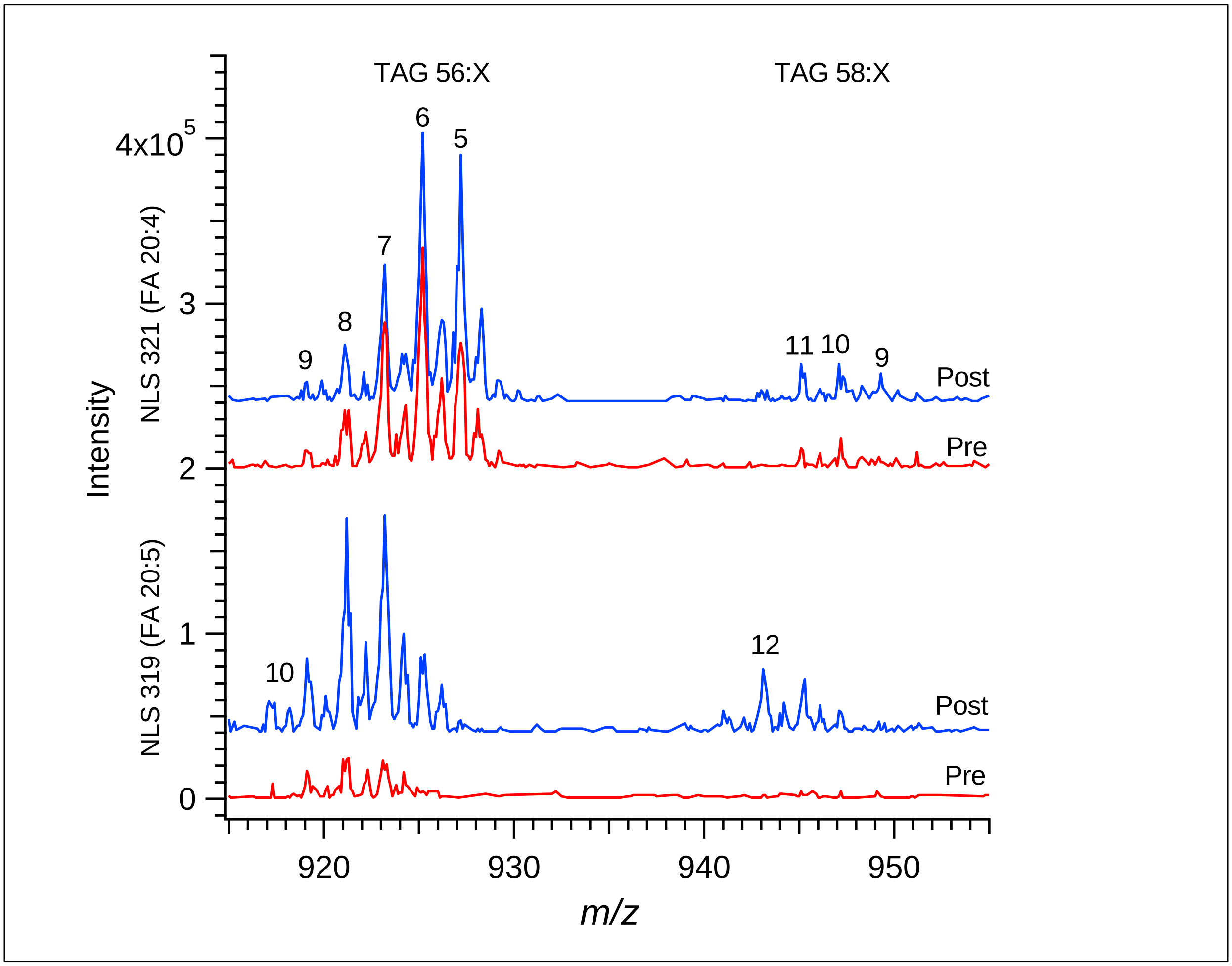

Figure S4. Identification of arachidonic acid (fatty acid (FA) 20:4)-containing plasma triacylglycerol (TAG) species using neutral loss scans (NLS) for pre- and post-treatment for positive responder, non-responder, and negative responder phenotypes.

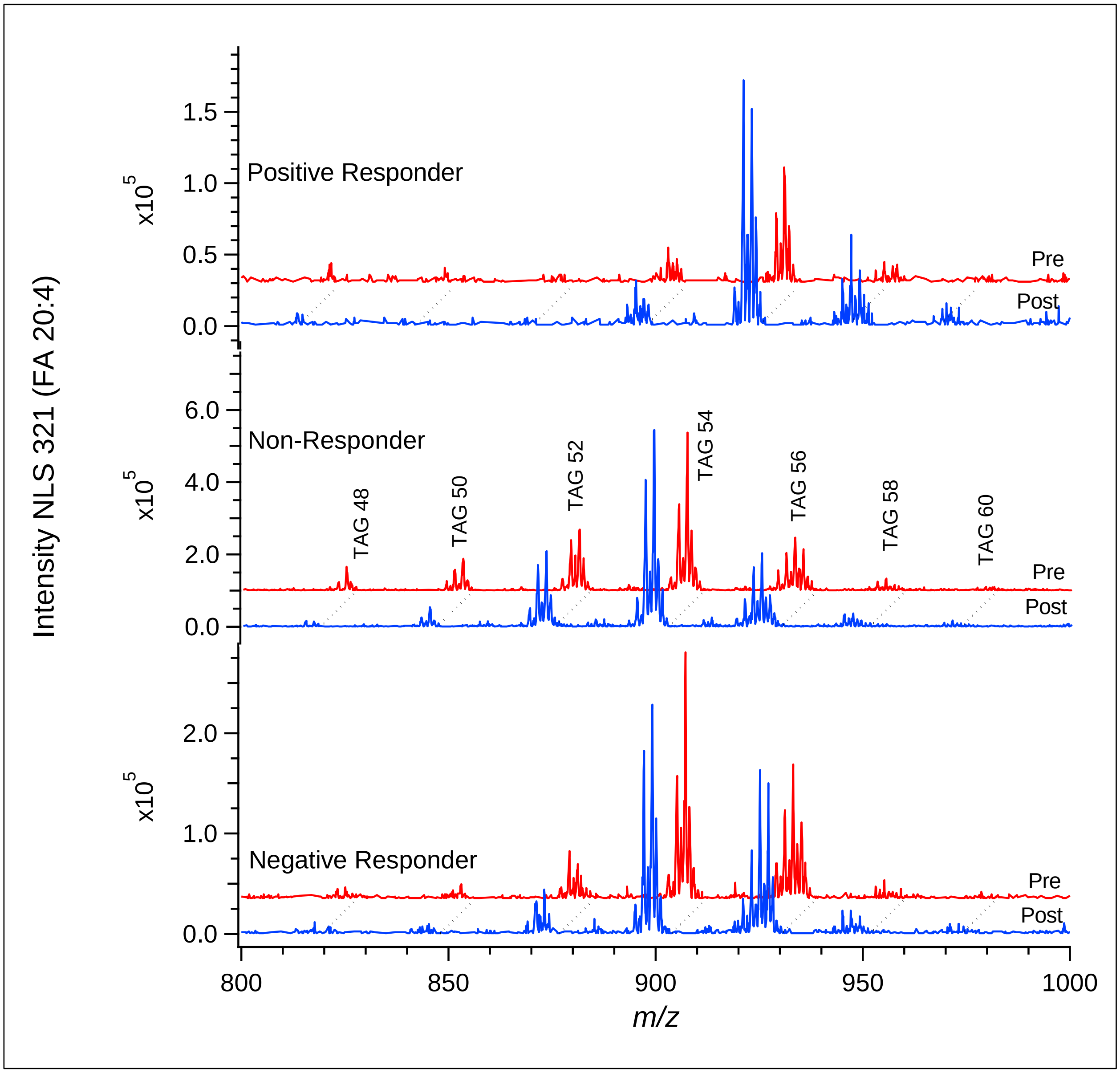

Figure S5. Identification of eicosapentaenoic acid (fatty acid (FA) 20:5)-containing plasma triacylglycerol (TAG) species using neutral loss scans (NLS) for pre- and post-treatment for positive responder, non-responder, and negative responder phenotypes.

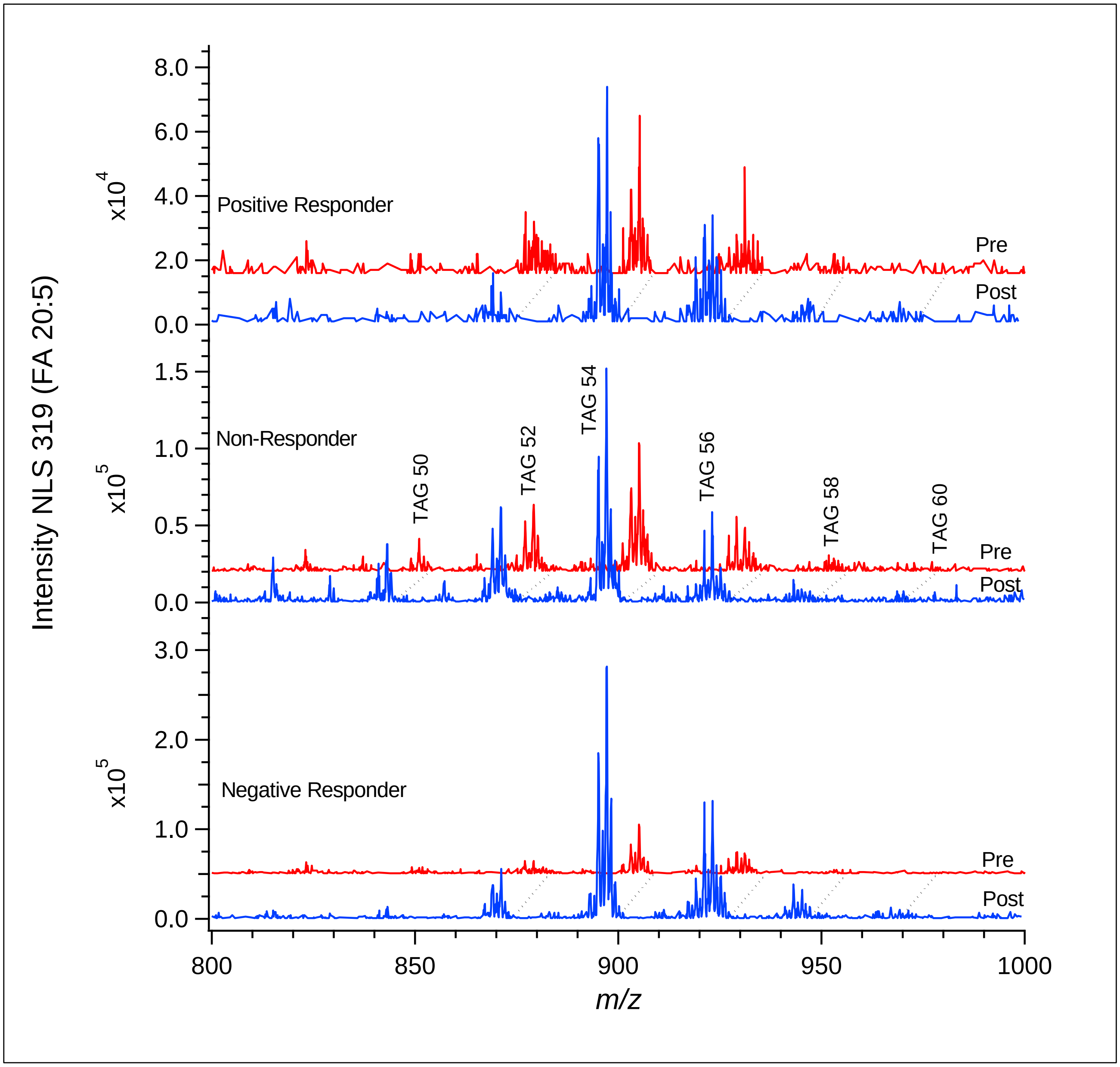

Figure S6. Identification of docosahexaenoic acid (fatty acid (FA) 22:6)-containing plasma triacylglycerol (TAG) species using neutral loss scans (NLS) for pre- and post-treatment for positive responder, non-responder, and negative responder phenotypes.

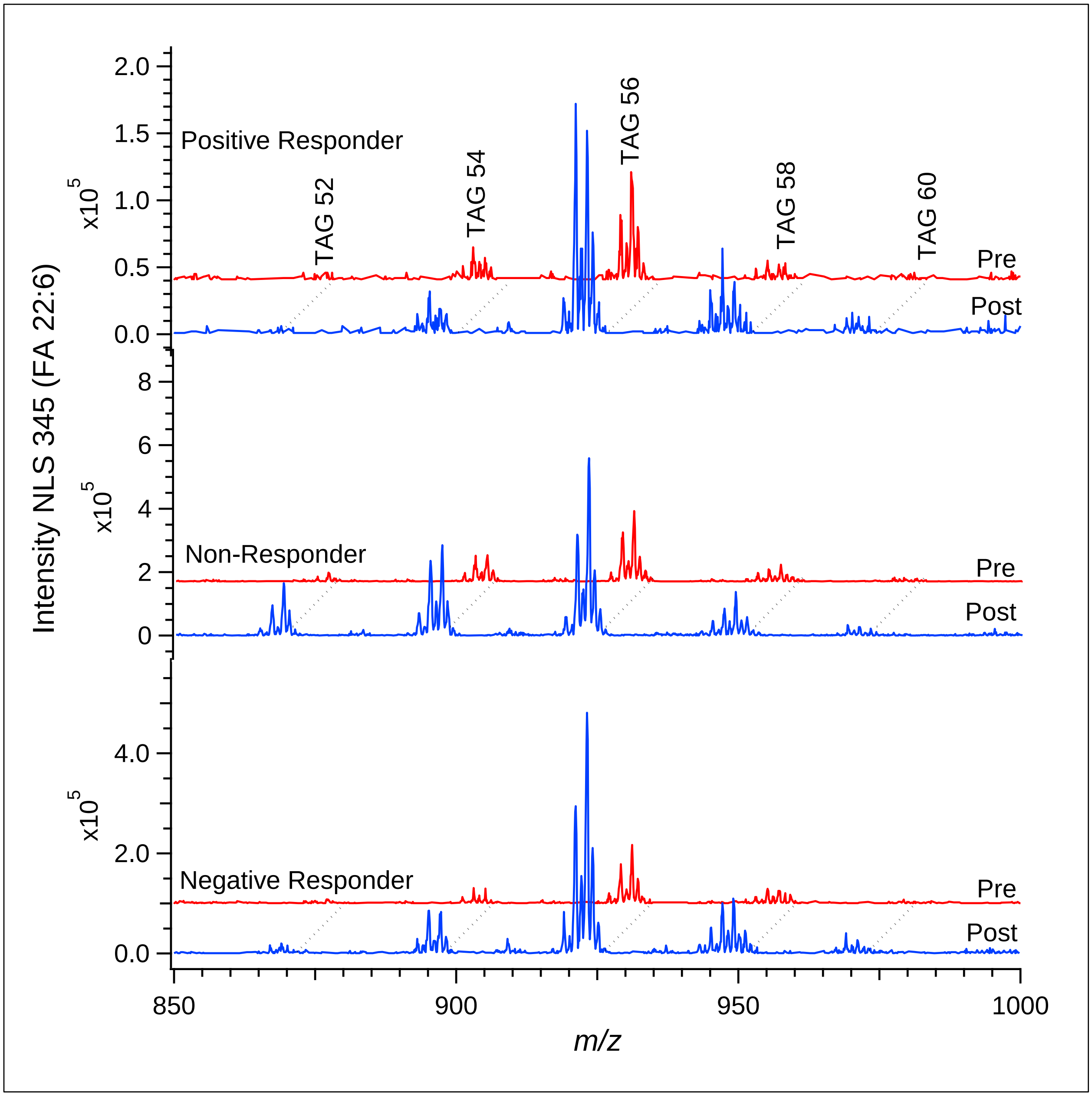

Figure S7. Product ion scans for the cholestane cation (m/z = 369) allow for the selective measurement of cholesteryl esters (CE). The internal standard CE 17:0 is indicated. Cholesteryl esters containing long-chain PUFA's are also indicated, with the most abundant species CE 18:2 shown for reference. Representative spectra are provided for the positive responder, non-responder, and negative responder phenotypes.

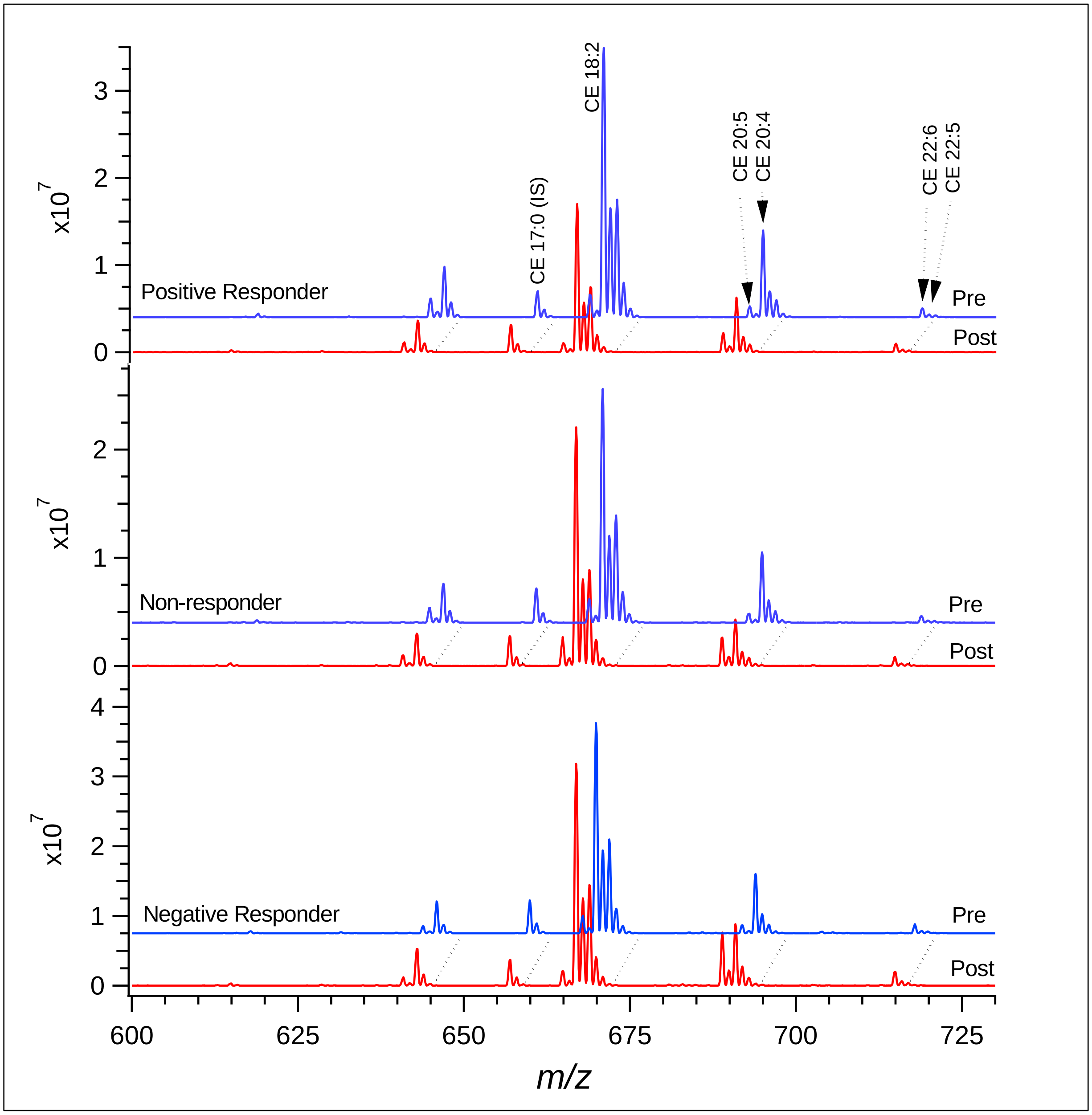

Figure S8. Identification of lauric acid (fatty acid (FA) 12:0)-containing plasma triacylglycerol (TAG) species using neutral loss scans (NLS) for pre- and post-treatment positive responder, non-responder, and negative responder phenotypes.

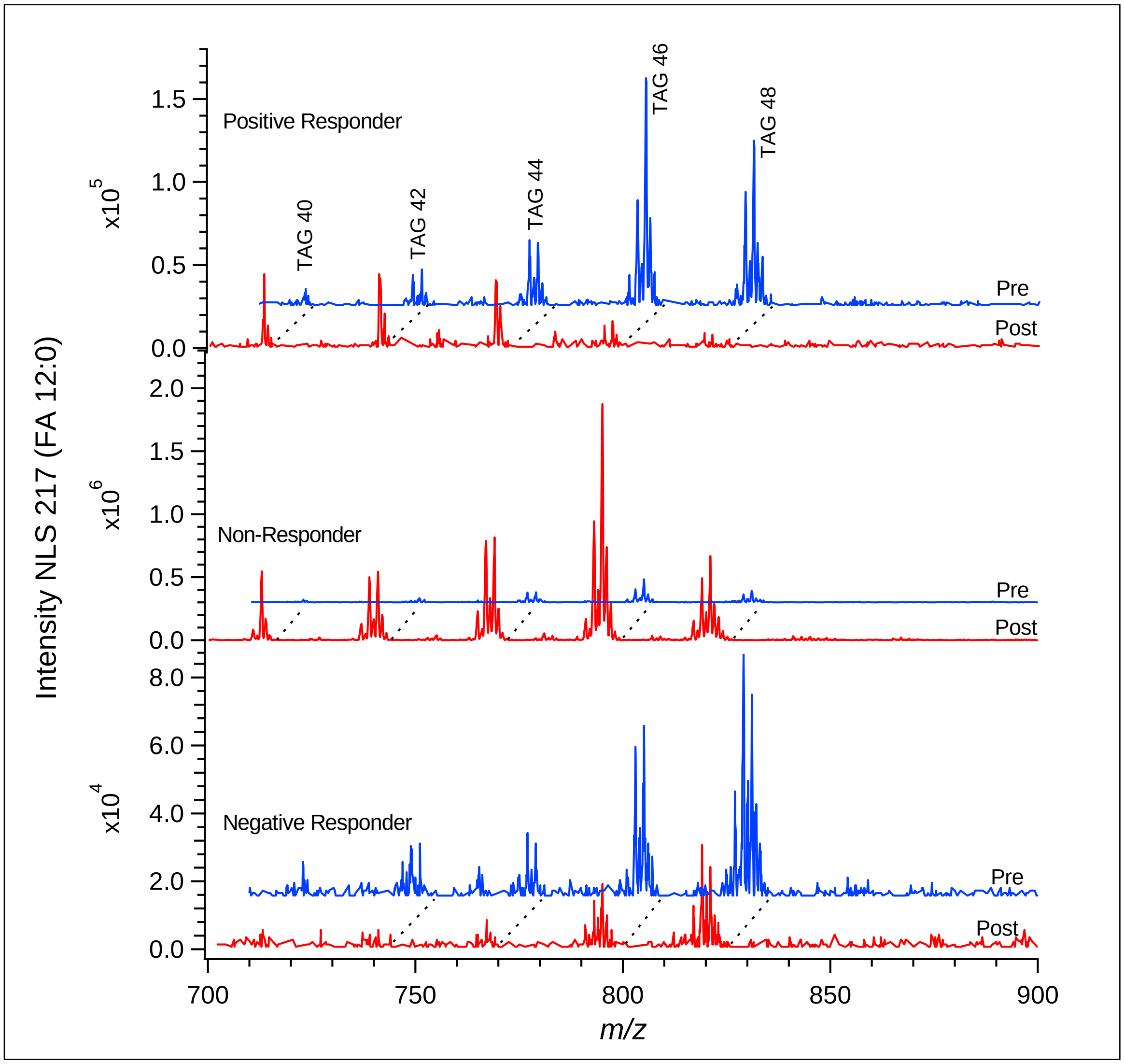

Figure S9. Identification of myristic acid (fatty acid (FA) 14:0)-containing plasma triacylglycerol (TAG) species using neutral loss scans (NLS) for pre- and post-treatment for positive responder, non-responder, and negative responder phenotypes.

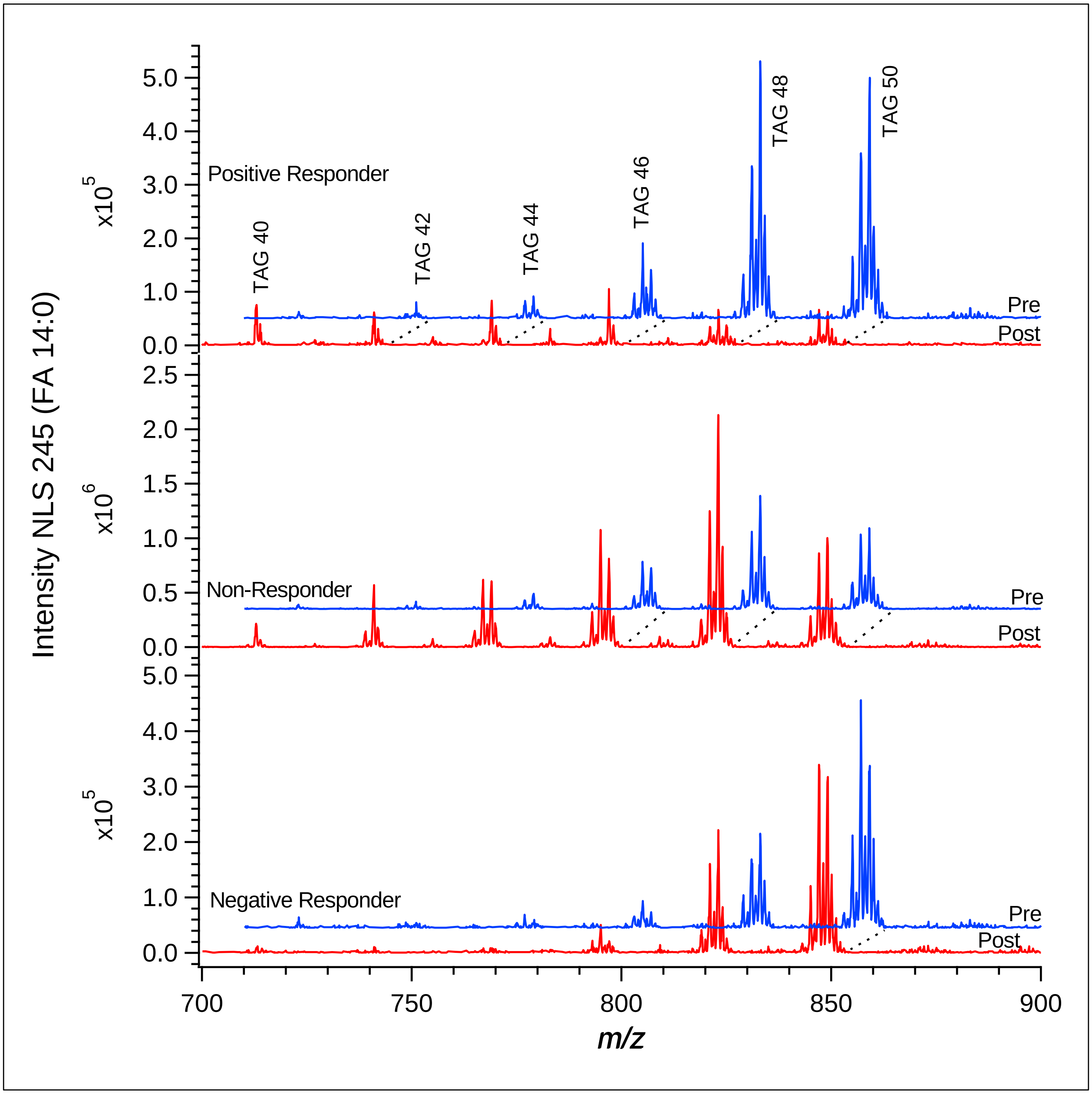

Supplement: Supplementary file 2 — Tables S1–S3 Figures S1–S9 [file JAH3-10-e018126-s002.pdf]
